# Brain structural pathways linking dietary patterns and cognitive performance in adolescents

**DOI:** 10.1007/s00429-026-03190-w

**Published:** 2026-08-29

**Authors:** Isabelle Brocas, Liam Kirwin, Matthew Kuloszewski

**Affiliations:** 1 University of Southern California, IAST and CEPR, Los Angeles, USA; 2 University of Southern California, Los Angeles, USA

**Keywords:** adolescent brain development, nutrition, cognitive performance, white matter, fractional anisotropy, fornix, ABCD Study

## Abstract

Dietary patterns covary with cognitive performance during adolescence, but the structural brain features associated with this covariance remain incompletely characterized. To identify structural brain correlates of cognition-aligned dietary patterns in adolescents while addressing adiposity, ethnicity adjustment, family relatedness, and multiple testing. We analyzed 3,308 adolescents from ABCD Release 5.1. Ten food-group measures were summarized by five dietary principal components (PCs). We constructed sample-derived, cognition-aligned dietary scores (PolyIQ) and used repeated five-fold family-level cross-fitting so that each participant’s score was calculated using cognition-derived weights estimated without that participant or members of the same family. The original PCs provided an outcome-independent benchmark. Primary models adjusted for age, sex, pubertal stage, socioeconomic status, income-to-needs ratio, sleep, and age- and sex-standardized BMI; ethnicity was excluded from primary models and added in sensitivity analyses. Brain–cognition models and family-cluster bootstrap indirect-effect models examined fractional anisotropy (FA), cortical thickness, surface area, and regional volume. All pre-specified indirect-effect models, including nonsignificant results, were retained. Cross-fitted dietary scores were associated with crystallized cognition (β = 0.099, SE = 0.018, *P* < 0.001) and fluid cognition (β = 0.064, SE = 0.015, *P* < 0.001); the five original dietary PCs were jointly associated with both domains. Brain–cognition associations in the bilateral fornix and hippocampal cingulum remained after BMI adjustment. In the all-ROI analysis, indirect associations through right and left fornix FA survived false-discovery-rate correction for both crystallized cognition (right: 0.0067, 95% CI [0.0016, 0.0134]; left: 0.0063, [0.0013, 0.0126]) and fluid cognition (right: 0.0061, [0.0019, 0.0127]; left: 0.0052, [0.0011, 0.0106]). No gray-matter indirect effect survived correction across all tested regions. A sample-derived dietary pattern aligned with cognition covaried with FA in bilateral fornix pathways. The findings were robust to adiposity adjustment and were similar when ethnicity was added, but the cross-sectional design supports only a decomposition of shared variance, not a causal or temporally ordered mechanism.

**Supplementary Information:**

The online version contains supplementary material available at https://doi.org/10.1007/s00429-026-03190-w.

## Introduction

Adolescence represents a period of extraordinary neuroplasticity characterized by continued structural and functional brain development that extends into the third decade of life (Blakemore 2008, 2012; Casey et al. 2018). During this critical developmental window, environmental factors are associated with variation in neural circuits supporting higher-order cognition (Giedd 2008; Fuhrmann et al. 2015). The adolescent brain undergoes substantial structural refinement, including protracted myelination of association tracts and synaptic pruning that shapes connectivity patterns within and between brain networks (Lebel and Beaulieu 2011; Tamnes et al. 2017). This extended period of neuroplasticity creates both vulnerability to adverse environmental influences and opportunities to study environmental correlates of cognitive development.

### Brain development and environmental sensitivity

Key brain structures supporting memory and executive function—including the hippocampus, parahippocampal cortex, and connecting white-matter (WM) tracts such as the fornix— undergo protracted maturation throughout adolescence (Tamnes et al. 2013; Krogsrud et al. 2016). The fornix is a WM pathway connecting the hippocampus to frontal and subcortical memory systems and contributes to episodic memory formation and flexible reasoning (Rudebeck et al. 2009). These memory-related circuits may be sensitive to environmental inputs during adolescence (Gómez-Pinilla 2008).

The parahippocampal cortex and temporal language regions similarly show continued structural development during adolescence while supporting contextual memory integration and semantic processing—functions relevant to crystallized cognition (Gogtay et al. 2004). This convergence of ongoing structural maturation and functional specialization creates a developmental context in which nutrition may covary with cognitive outcomes and their neural correlates.

### Nutrition and neurodevelopment

Emerging evidence suggests that nutritional quality during adolescence is associated with markers of brain development through multiple biological pathways. For instance, high sugar intake promotes neuroinflammation and impairs synaptic plasticity, particularly in memory-related regions (Kanoski and Davidson 2011; Beilharz et al. 2015).

Current research indicates that healthier dietary patterns are associated with better cognitive performance in youth (Cohen et al. 2016), with Mediterranean-style diets correlating with executive function and academic achievement (Esteban-Cornejo et al. 2016).

Most studies, however, use isolated foods, nutrients, or externally defined pattern scores. These approaches may not provide a single, transparent summary of the distributed dietary variation most closely aligned with a particular cognitive domain.

### The present study

Understanding the neurobiological features that co-vary with both nutrition and cognitive performance during adolescence is particularly urgent given stark nutritional disparities among youth. Only 7% of US adolescents meet federal dietary guidelines, with significant gaps by socioeconomic status (Rehm et al. 2016; Banfield et al. 2016). Moreover, emerging evidence suggests that nutritional effects on cognition may be strongest among disadvantaged populations (Parrott et al. 2021), indicating that dietary interventions could simultaneously promote optimal brain development and reduce educational disparities.

Here we examine concurrent associations among diet, cognition, and structural brain measures in a large adolescent sample from the ABCD Study. Diet is represented in two complementary ways. First, unsupervised PCA summarizes ten correlated food-group measures into five orthogonal dietary dimensions. These PCs provide the outcome-independent representation of diet and permit direct joint tests of the dietary block. Second, we use the term PolyIQ for a supervised, sample-derived dietary index that combines the same PCs according to their association with either crystallized or fluid cognition. PolyIQ is calculated entirely from dietary variables, but its weights are outcome-specific; it is therefore a cognition-aligned dietary pattern score rather than a normative measure of diet quality or an independently validated exposure. To address reuse of the same cognitive observations in score construction and downstream analyses, we use repeated family-level cross-fitting. Each participant’s PolyIQ score is generated from weights estimated in other families. This preserves the scientific purpose of identifying the dietary direction most relevant to cognition while preventing a participant’s own cognitive outcome, or that of a sibling, from determining the score subsequently used for that participant. We report the original PCs alongside PolyIQ, and we treat the direct PolyIQ–cognition regression as an out-of-fold characterization of the supervised dietary signal rather than as evidence that a newly discovered independent exposure predicts cognition.

We then ask whether this cognition-aligned dietary direction covaries with structural brain measures and whether individual brain measures statistically account for part of that covariance. Brain data are not used to construct PolyIQ, so the diet–brain component of the analysis introduces information not mechanically contained in score construction. Nevertheless, all three domains were measured concurrently. We therefore use directional notation only as a statistical parameterization and interpret indirect effects as decompositions of shared variance rather than causal mediation. This approach offers several advantages for the present study. It preserves the multidimensional nature of diet, avoids focusing on isolated foods or nutrients, and provides a compact way to examine whether the overall pattern of diet–cognition covariation is statistically linked to brain structure. At the same time, PolyIQ remains exploratory and should not be interpreted as an independent predictor of cognition, a normative index of dietary quality, or a causal measure of nutritional influence.

## Methods

### Participants and analytic samples

We used ABCD Annual Release 5.1, available through the NIMH Data Archive (DOI: https://doi.org/10.15154/z563-zd24). ABCD recruited 11,878 children aged 9–10 years across 21 US sites (Garavan et al. 2018; Volkow et al. 2018). The present cross-sectional analysis used the four-year follow-up because it was the only wave in Release 5.1 with overlapping nutrition, cognition, and neuroimaging measures. We further avoided relying on later data releases, as additional participants may have experienced longer-term effects of the COVID-19 pandemic. In Release 5.1, most assessments were conducted in 2021, with a marked drop in December 2021, potentially reflecting disruptions related to the surge in cases accompanying the rapid emergence and spread of the Omicron variant (Supplementary Methods, Sect. 1.1). The main merged sample contained 3,308 participants. Imaging availability and complete covariate coverage varied by modality (Table 1).

**Table 1:** Analytic sample sizes

Analysis	Available	Primary model
Main diet and cognition sample	3,308	2,145
FA	1,520	1,423
Surface area	2,043	1,774
Cortical thickness, named ROIs	2,013	1,743
Regional volume	1,854	1,611

The primary complete-case models include age- and sex-standardized BMI and the other pre-specified covariates. The named-ROI cortical-thickness file contains 2,013 participants; this corrects the earlier reported value of 1,644, which referred to a different thickness extract with unmapped variable names

All variables used in the primary analyses were treated as concurrent measures from the same follow-up wave. Accordingly, all analyses are cross-sectional and should be interpreted as characterizing shared variance among diet, brain structure, and cognition rather than temporal or causal pathways.

In the main sample, 2,340 children belonged to singleton families and 968 belonged to 479 multi-child families. Cognition models used a family random intercept. For imaging models, site was included as a fixed effect. The FA analytic sample contained no repeated families after complete-case restriction, so a family random intercept was not identifiable there; other modality-specific analyses used family-cluster bootstrap inference or mixedeffects models when repeated families were present. Details about sample structure and missingness are reported in Supplementary Methods, Sect. 1.2.

### Representativeness of the synthetic sample and COVID impact

Compared to the full ABCD cohort initially recruited (11,878 children at baseline), our main synthetic sample excludes participants for which either cognitive or nutrition data are missing. In ABCD Release 5.1, Year 4 data are available only for a subset of participants because ABCD releases are cumulative snapshots based on a fixed data-freeze date; accordingly, later follow-up waves are only partially complete at the time of release. The partial availability of Year 4 data in ABCD Release 5 also reflects disruptions associated with the COVID-19 pandemic, during which in-person visits were suspended and some protocol elements were administered remotely or in hybrid form, while others such as neuroimaging still required in-person assessment.

Year 4 assessments in ABCD occurred during the COVID era. Accordingly, we do not interpret Release 5 Year 4 data as unaffected by the pandemic. Rather, our view is that Release 5, which contains only interim Year 4 data, likely reflects a narrower and earlier segment of the Year 4 assessment window than later releases in which Year 4 is complete. To the extent that pandemic-related disruptions and subsequent recovery altered dietary behaviors over calendar time, later releases may therefore introduce greater heterogeneity in the timing and context of nutrition measurement.

For transparency, we provide descriptive comparisons between included vs. excluded participants in the main demographic variables in Supplementary Methods, Sect. 1.4, Table S5. This analysis indicates that the analytic sample is not fully representative of the ABCD cohort and this may limit generalizability.

### Dietary assessment and preprocessing

Dietary intake was assessed using the Block Kids Food Screener (BKFS), a brief instrument designed to estimate usual dietary intake in children and adolescents (Cullen et al. 2008). We focused on food-group variables capturing broad aspects of dietary patterning rather than isolated nutrients. The variables entered into the dietary pattern analysis were fruits (excluding juice), vegetables (non-potato), whole grains, dairy products, meat/poultry/fish, legumes and nuts, potatoes (including fries), soft drinks/sodas, sugar-sweetened beverages, and condiments (e.g., ketchup, salsa). To limit the influence of outliers, these variables were winsorized at the 1st and 99th percentiles. The resulting variables were then logtransformed using log(1 + x) to address skewness and were subsequently standardized into z-scores. Missingness was low for dietary variables (2.1%). Supplementary descriptive statistics for these variables are reported in Supplementary Methods, Sect. 1.3. We chose a dietary-pattern approach because the research question concerned multivariate eating habits rather than the isolated effect of any single food item or nutrient. This approach also reduces dimensionality and captures combinations of foods that tend to be consumed together.

### Cognitive outcomes

The two outcomes were study-specific composites constructed from NIH Toolbox Cognition Battery (Weintraub et al. 2013) administered in the ABCD Study (ABCD Study data documentation, Neurocognition page, version 6.1.3, https://doi.org/10.5281/zenodo.15800725), including tasks measuring vocabulary, reading, episodic memory, working memory, processing speed, attention, inhibitory control, and cognitive flexibility. Crystallized cognition was the mean of standardized Oral Reading Recognition and Picture Vocabulary scores. Fluid cognition was the mean of standardized Flanker Inhibitory Control and Attention, List Sorting Working Memory, and Picture Sequence/Episodic Memory scores.

Cognitive missingness was modest for Reading, Picture Vocabulary, List Sorting, and Episodic Memory (approximately 1.7%–2.3%), but higher for Flanker (28.7%), whereas Card Sort was missing for nearly all participants (about 97.6%). We therefore excluded Card Sort and retained the five tasks with sufficient coverage to construct crystallized and fluid cognition composites. Missing values in these retained tasks were addressed using predictive mean matching implemented in the mice package from R, with the imputation model including the five cognitive measures as well as age, sex, social economic status (SES), pubertal stage, sleep duration, sleep efficiency, and social jetlag. This approach was selected because it preserves plausible observed values and reduces the risk of implausible model-based imputations. Composite cognition scores were then reconstructed from the imputed component measures using the same task structure as the ABCD-derived scores. In the present study, both outcomes were analyzed as standardized scores. Supplementary descriptive statistics for these composites are reported in Supplementary Methods, Sect. 1.5.

### Dietary PCA and PolyIQ construction

PCA was performed on the correlation matrix of the standardized food variables. Five PCs were retained using the scree plot, cumulative explained variance, and interpretability; together they explained 77.0% of dietary variance. The strict Kaiser rule would retain two PCs, but PCs 3–5 represented smaller interpretable dimensions that were retained for the prespecified multivariate dietary representation.

Although factor analysis could also have been used to identify latent dietary constructs, PCA was better suited to our aim of reducing a large set of correlated dietary indicators into a parsimonious set of empirical pattern scores for use in subsequent analyses.

The original PolyIQ score was a weighted sum of the five PCs, with weights estimated separately for crystallized and fluid cognition using elastic net (α = 0.5) and 10-fold crossvalidation for λ. Because the PCs are orthogonal, penalization was used as a conservative score-weighting device rather than as a remedy for multicollinearity. Scores based on ordinary least squares were almost identical to the elastic-net scores (*r* > 0.999 in both domains).

For inferential analyses, PolyIQ was reconstructed by repeated five-fold family-level cross-fitting. Entire families were assigned to folds. Within each outer training sample, λ was selected by inner family-level cross-validation, weights were estimated using only the training families, and the weighted PC score was calculated for the held-out families. Heldout scores were standardized within each repeat and averaged across ten repeats. Thus no participant’s cognition, and no sibling’s cognition, contributed to the weights used to calculate that participant’s downstream score.

### Neuroimaging measures

We analyzed ABCD-distributed derived summary measures rather than reprocessing raw images. After first defining fractional anisotropy, we use FA throughout. The seven WM measures were bilateral fornix, bilateral hippocampal cingulum, bilateral inferior longitudinal fasciculus, and forceps major. Gray-matter (GM) measures included prespecified regional volume, cortical thickness, and surface-area ROIs in memory, language, and executive-control systems. Supplementary Methods, Sect. 1.6. lists every tested ROI and details acquisition, FreeSurfer processing, diffusion preprocessing, parcellation, and quality control. Regional volume models adjusted for intracranial volume, surface-area models for total cortical area, and thickness models for mean cortical thickness. No global size adjustment was used for FA.

Imaging values were not imputed. We also utilized the ABCD derived ”inclusion” variables and excluded all images marked as 0. Neuroimaging analyses were conducted using measure-specific complete-case samples because MRI missingness was not assumed to be missing completely at random.

### Covariates and sensitivity specifications

Primary models included age, sex, pubertal stage, SES, income-to-needs-ratio (INR), sleep duration, sleep efficiency, social jetlag, and age- and sex-standardized BMI (BMI SDS). SES was modeled as a composite measure provided by the ABCD dataset, whereas INR captured household economic resources relative to family size. These covariates were selected because they are plausibly associated with both dietary behavior and cognitive performance during adolescence. Anthropometric quality control excluded six implausible complete height/weight records; BMI SDS was available for 2,444 participants after quality control. Ethnicity was excluded from primary inferential models and retained for descriptive reporting. To conduct ethnic-group sensitivity analyses, otherwise identical models including the ABCD race/ethnicity variable were also reported. Additional specifications omitted adiposity or included both BMI SDS and standardized waist circumference. Covariate specifications are described in Supplementary Methods 1.7.

### Timing of data collection

In the ABCD protocol, dietary questionnaires, neurocognitive testing, and MRI are collected as components of the broader assessment visit structure. However, based on publicly available ABCD documentation, we did not identify evidence of a fixed universal ordering of the BKFS nutrition questionnaire relative to cognitive testing and MRI across visits/sites.

At the 4-year follow-up, we merged the cognition, MRI, and nutrition files and restricted the sample to children with all three assessments available. For each child, we ordered the assessments chronologically and computed the intervals in days between consecutive tests. The most common assessment sequence was Cognition →MRI →Nutrition (*n* = 2,549), followed by MRI →Nutrition →Cognition (*n* = 298), while all other orders were relatively uncommon. Across all consecutive test pairs, the median interval was 0 days, indicating that many assessments occurred on the same day, although the mean interval was 9.29 days due to a small number of very large gaps. Transition-specific summaries showed that MRI and nutrition were almost always administered on the same day (MRI →Nutrition: mean = 0.002 days, median = 0), whereas cognition was more often separated in time from the other assessments, particularly relative to MRI (Cognition →MRI: mean = 19.5 days, median = 1). Overall, these results suggest a strongly patterned scheduling structure in which cognition typically occurs first and MRI and nutrition are commonly clustered together.

### Statistical analysis

The analysis proceeded as follows. First, we described the dietary PCs and PolyIQ weights. Second, we estimated mixed-effects cognition models using cross-fitted PolyIQ and separately tested the joint contribution of all five original PCs by likelihood-ratio tests. Third, brain–cognition associations were estimated under four prespecified covariate specifications: no adiposity/no ethnicity; BMI SDS/no ethnicity (primary); BMI SDS plus waist/no ethnicity; and BMI SDS plus ethnicity. Fourth, all prespecified brain ROIs were entered into family-cluster bootstrap path models using cross-fitted PolyIQ, rather than restricting reporting to significant screening results. The mediator model estimated the association of diet with the brain measure, and the outcome model included both diet and the brain measure. We report the product-of-coefficients indirect association, direct association, total association, and proportion accounted for, with 1,000 family-cluster bootstrap replications. Benjamini–Hochberg FDR correction was applied separately within each modality, cognitive outcome, and effect family. The complete set of tested indirect effects, including nonsignificant effects, is reported in the supplement. PC-based FA models treated each PC as the focal exposure while controlling for the other four PCs and corrected across all PC-by-tract tests.

*Software.* All analyses were conducted in R version 4.2.0. Aside from base R packages, we used texreg, ggplot2, DiagrammeR, DiagrammeRsvg and rsvg to create tables, figures and diagrams and Python nilearn.plotting to create illustrations of the brain components involved in the analysis.

## Results

### Dietary PCs and cognition-aligned score construction

**Fig. 1:**
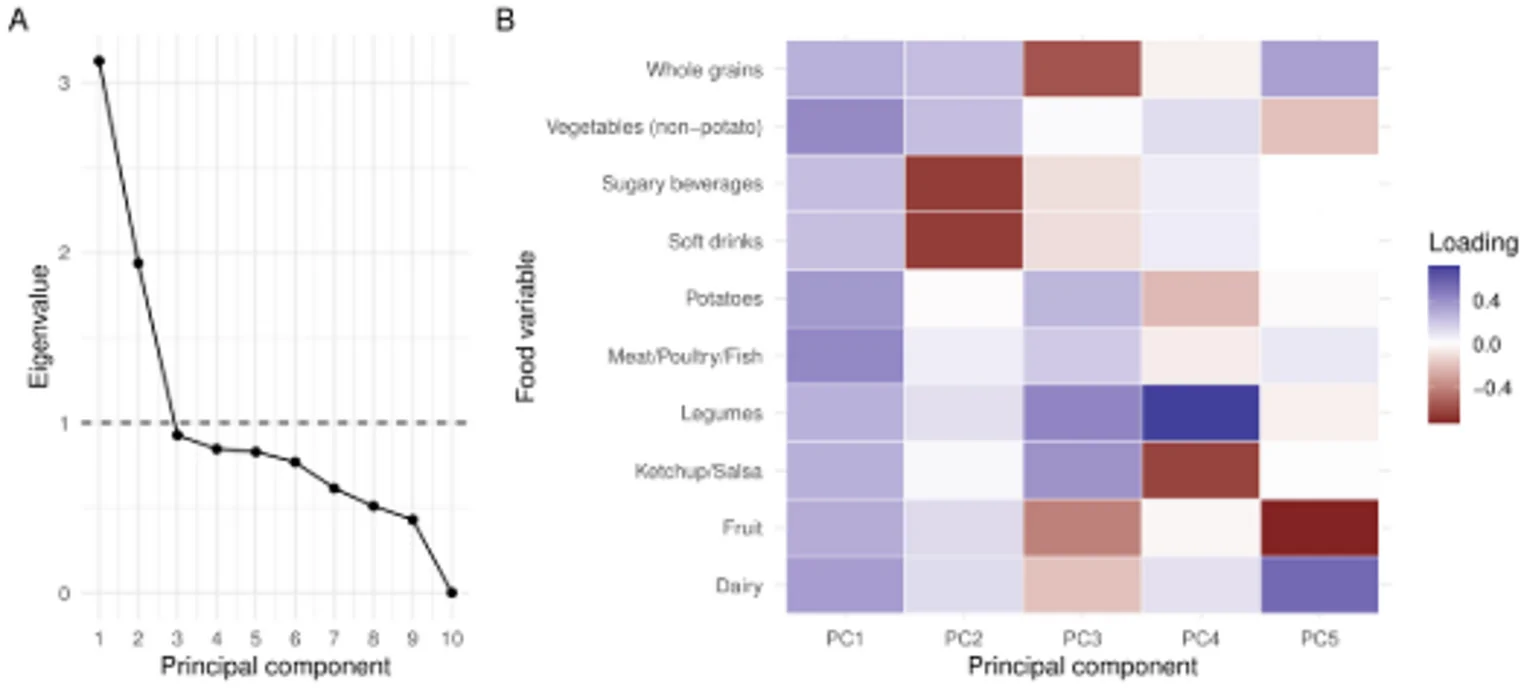
Food-pattern PCA. The left panel shows the scree plot and the right panel shows loadings for the five retained dietary PCs

The first five dietary PCs explained 77.0% of dietary variance (Fig. 1). PC1 represented broadly higher consumption across food groups, PC2 was dominated by lower soft-drink and sugar-sweetened-beverage intake, and PCs 3–5 captured smaller contrasts involving whole grains, legumes, condiments, dairy, and fruit.

**Fig. 2:**
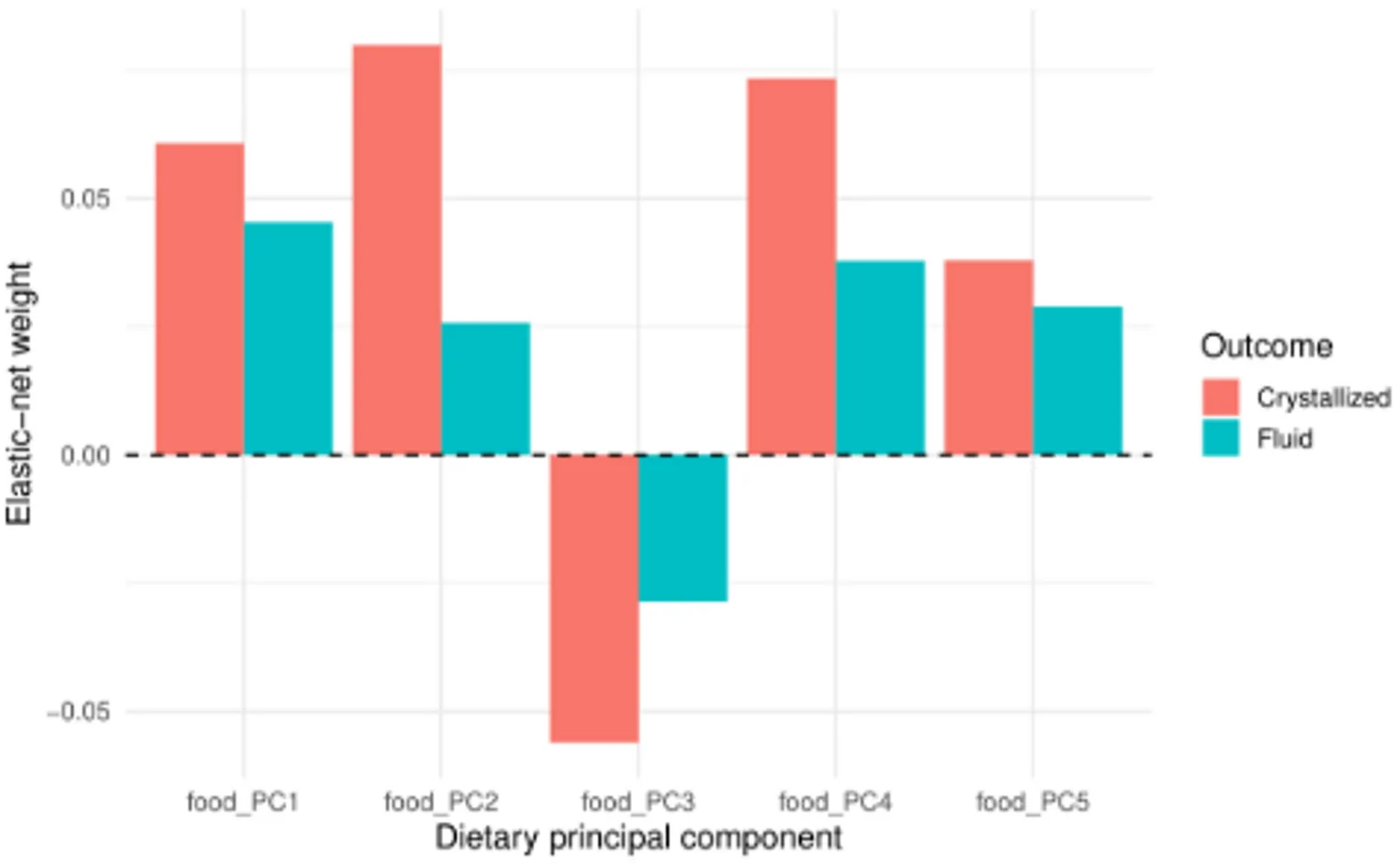
Elastic-net weights used to construct the sample-derived cognition-aligned dietary scores

The elastic-net weights are shown in Fig. 2. For crystallized cognition, the largest positive weights were PC2 and PC4, followed by PC1; PC3 received a negative weight. Fluid-cognition weights showed the same ordering with smaller magnitudes. Elastic-net and ordinary-regression scores were essentially identical (*r* = 0.99 in both domains). Details of these analyses are reported in Supplementary Results Sects. 2.1 and 2.2.

### Cross-fitted dietary scores and cognitive composites

The cross-fitted and in-sample scores were nearly identical (*r* = 0.9996 for crystallized and *r* = 0.9989 for fluid), while their correlations with the outcomes were slightly smaller under cross-fitting, as expected. In the primary BMI-SDS-adjusted models without ethnicity, a one-standard-deviation increase in the cross-fitted cognition-aligned dietary score was associated with a 0.099-SD higher crystallized composite and a 0.064-SD higher fluid composite (Table 2). Both associations were similar when adiposity was omitted, waist circumference was added, or ethnicity was included. Complete-case cognitive composites also produced similar estimates. Details are reported in Supplementary Results Sects. 2.2 and 2.3.

**Table 2:** Cross-fitted dietary-score associations and joint dietary-PC tests

Outcome/model	Estimate	SE	*P*	*N*
Panel A. Primary cross-fitted PolyIQ specification				
Crystallized cognition	0.099	0.018	< 0.001	2145
Fluid cognition	0.064	0.015	< 0.001	2145
Panel B. Joint test of all five original dietary PCs				
Crystallized cognition	χ² = 36.58	5 df	< 0.001	2145
Fluid cognition	χ² = 27.31	5 df	< 0.001	2145

Entries in Panel A are mixed-effects regression coefficients; the adjacent values are standard errors (SE). Models include a family random intercept and adjust for age, sex, pubertal stage, SES, INR, sleep duration, sleep efficiency, social jetlag, and BMI SDS. Ethnicity is excluded from the primary specification. Panel B reports likelihood-ratio tests comparing models with and without the five-PC dietary block

These results should not be interpreted as independent validation of PolyIQ as a new exposure. Rather, they show that the outcome-aligned dietary direction generalizes to heldout families and that the same conclusion is supported by an outcome-independent joint test of the original PCs.

### Brain–cognition associations with adiposity adjustment

In the primary BMI-SDS-adjusted specification, FA in both fornices and both hippocampal cingulum tracts was positively associated with crystallized cognition after FDR correction. Bilateral fornix FA and right hippocampal cingulum FA were associated with fluid cognition; left hippocampal cingulum FA was also significant in the primary specification but not after ethnicity was added. Left inferior longitudinal fasciculus FA was associated with crystallized cognition, and forceps major FA with fluid cognition. These coefficients were not attenuated by BMI adjustment and remained similar when waist circumference or ethnicity was added. Regional-volume associations surviving FDR correction were concentrated in crystallized cognition and included bilateral parahippocampal gyri, left pars orbitalis, bilateral fusiform regions, right middle temporal gyrus, left rostral middle frontal gyrus, and right precuneus. No regional-volume association with fluid cognition survived correction in the primary BMI-SDS model. Surface-area and cortical-thickness associations did not survive their modality-specific corrections. Full coefficients, standard errors, test statistics, and adjusted P values are reported in Supplementary Results Sect. 2.4.

### All-ROI indirect-effect analyses

**Fig. 3:**
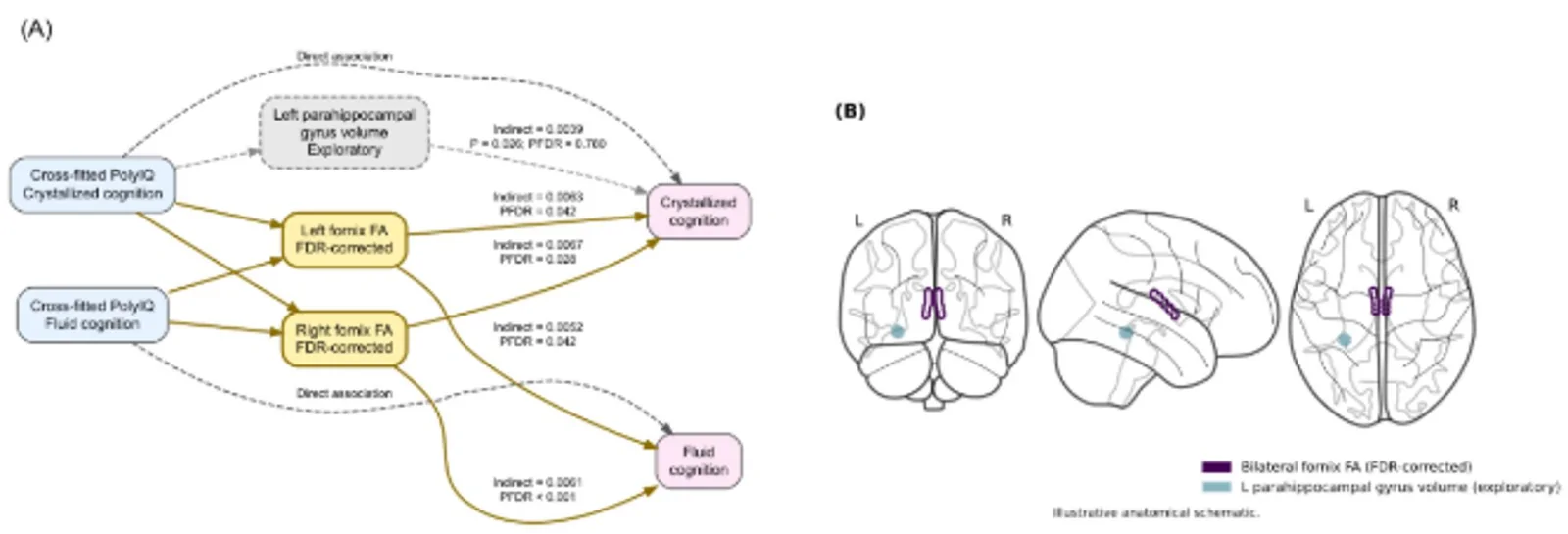
Indirect associations between cognition-aligned dietary patterns, brain structure, and cognitive performance. **A** Path diagram summarizing the revised analyses using cross-fitted cognition-aligned dietary scores. Solid paths indicate indirect associations that survived false-discovery-rate (FDR) correction, whereas dashed paths indicate exploratory associations that did not survive correction. **B** Anatomical illustration of the brain measures implicated in these analyses. Bilateral fornix fractional anisotropy (FA; purple) showed FDR-corrected indirect associations with both crystallized and fluid cognition. Left parahippocampal gyrus volume (blue) showed an exploratory indirect association with crystallized cognition that was significant before, but not after, correction for multiple comparisons. Anatomical locations are shown for illustration and do not represent voxelwise statistical coordinates. *L* left hemisphere, *R* right hemisphere

The all-ROI family-cluster bootstrap analysis identified four FDR-corrected indirect associations, all involving bilateral fornix FA (Table 3; Fig. 3). Right and left fornix FA accounted for approximately 6.4% and 6.1%, respectively, of the cross-fitted dietary-score association with crystallized cognition. For fluid cognition, the corresponding proportions were approximately 9.7 and 8.2%. These proportions are descriptive decompositions of concurrent covariance. Figure 3 illustrates the results.

**Table 3:** FDR-significant indirect associations in the all-ROI analysis

Outcome	Mediator	Indirect estimate	95% CI	*P*	P_FDR
Crystallized	Right fornix FA	0.0067	[0.0016, 0.0134]	0.004	0.028
Crystallized	Left fornix FA	0.0063	[0.0013, 0.0126]	0.012	0.042
Fluid	Right fornix FA	0.0061	[0.0019, 0.0127]	< 0.001	< 0.001
Fluid	Left fornix FA	0.0052	[0.0011, 0.0106]	0.012	0.042

Estimates are products of the diet–brain and brain–cognition coefficients from models using cross-fitted PolyIQ and 1,000 family-cluster bootstrap replications. Primary models adjust for BMI SDS and exclude ethnicity. FDR correction was performed across all tested mediators within modality, cognitive outcome, and effect family

No GM indirect effect survived the complete all-ROI correction. Left parahippocampal volume showed a small uncorrected indirect association with crystallized cognition (estimate = 0.0039, 95% CI [0.0003, 0.0091], uncorrected *P* = 0.026), but its FDR-adjusted value across the 30 volume tests was 0.780. Targeted sensitivity models produced similar small estimates when adiposity was omitted, waist was added, or ethnicity was included; these analyses are therefore presented as secondary rather than confirmatory (see Supplementary Results, Sects. 2.5 and 2.6).

### PC-based and direct diet–brain robustness

When each original PC was tested while controlling for the other four, the indirect association PC1 →right fornix FA →fluid cognition survived correction across all PC-bytract tests (estimate = 0.0052, 95% CI [0.0014, 0.0104], adjusted *P* < 0.001). The corresponding crystallized-cognition effect was positive but did not survive the broad correction (PFDR = 0.070). This PC result provides an outcome-independent dietary counterpart to the cross-fitted PolyIQ analysis.

In direct diet–brain models, PC1 was positively associated with right fornix FA after FDR correction (β = 0.0010, PFDR = 0.030). No individual raw food variable survived correction for right fornix FA. Likewise, no food PC or individual food variable survived correction for left parahippocampal volume. Deatils are reported in Supplementary Results, Sects. 2.7 and 2.8.

### Supplementary robustness and exploratory analyses

Several additional analyses were conducted to assess the robustness and scope of the diet– cognition associations and are reported in the Supplementary Additional Analyses, Sect. 3. First, we examined whether the association between the cross-fitted cognition-aligned dietary scores and cognitive performance varied with social jetlag, pubertal stage, socioeconomic status (SES), or income-to-needs ratio (INR). These moderation models used the same primary covariate specification as the main analyses. Second, because SES and INR were independently associated with cognitive performance, we examined whether these socioeconomic associations were accompanied by statistical indirect associations through the cross-fitted cognition-aligned dietary scores. Given the cross-sectional design, these analyses are interpreted as decompositions of shared covariance rather than as evidence of causal mediation. As an outcome-independent robustness check, we repeated these analyses using each of the five unsupervised dietary principal components in place of PolyIQ. Because the dietary PCs were constructed solely from dietary variables, these models provide a complementary assessment that does not depend on cognition-informed weighting of the dietary exposure.

Finally, we examined whether the results depended on representing diet through food categories by conducting a separate PCA based on nutrient-level measures, including macronutrients, energy intake, sugars, saturated fat, glycemic index, and glycemic load. We characterized the resulting nutrient components and repeated the principal nutrition–cognition analyses using this alternative representation of dietary variation. Full methods, estimates, multiple-comparison corrections, and sensitivity analyses are reported in the Supplementary Material Sect. 3. These supplementary analyses showed a significant moderation of the diet–fluid cognition association by INR, significant socioeconomic indirect associations through the crossfitted dietary scores for both cognitive domains, and convergent outcome-independent evidence using the original dietary PCs, particularly PC1 and, for crystallized cognition, PC2 (see Supplementary Material, Sect. 3).

## Discussion

The analysis distinguishes three questions. The PCA describes dietary variation without using cognition. PolyIQ defines a supervised dietary direction aligned with a particular cognitive composite. Cross-fitting then evaluates that direction in held-out families and permits downstream brain analyses without reusing an individual’s own cognition in the score applied to that individual. The direct PolyIQ–cognition coefficient is therefore not presented as discovery of an independent dietary exposure; it is an out-of-fold characterization of a supervised score. The joint PC tests and PC-based mediation provide the outcome-independent benchmarks.

The most consistent structural result concerns the fornix. Brain–cognition associations for bilateral fornix FA remained after BMI-SDS adjustment, were not materially changed by adding waist circumference, and persisted when ethnicity was included as a sensitivity covariate. In the all-ROI analysis, both left and right fornix FA statistically accounted for small portions of the dietary-score covariance with both cognitive domains. The PC-based analysis further showed that PC1 accounted for an indirect association through right fornix FA for fluid cognition after correction across all PC-by-tract tests. Together, these findings suggest that the distributed dietary pattern captured by the dominant food PC and by the supervised score is selectively related to a hippocampal output pathway. The GM conclusion is conservative. Several regional volumes were associated with crystallized cognition after adiposity adjustment, but no GM indirect effect survived correction across all tested regions. The left parahippocampal result was small, individually significant in some targeted specifications, and consistent in sign, yet its all-volume FDR-adjusted result was null. It should therefore be viewed as a candidate for future longitudinal work rather than evidence comparable to the bilateral fornix findings.

Adiposity does not explain the principal WM findings. Adjusting for BMI SDS left the fornix and cingulum coefficients similar or slightly larger, and adding waist circumference did not change the qualitative pattern. Ethnicity was excluded from the primary inferential specification but included in sensitivity analyses. The core bilateral fornix conclusions remain. Several limitations should temper interpretation. All variables are concurrent, so directional path models cannot establish temporal precedence, causality, or whether diet affects brain structure rather than the reverse. PolyIQ remains sample-derived and outcomespecific even under cross-fitting; external validation in another cohort would be required before it could be used as a general dietary score. The primary cognitive composites use predictive-mean-matching values from one completed dataset, although complete-case crossfitted analyses were similar; future work should propagate imputation uncertainty through the full cross-fitting and bootstrap pipeline. Dietary intake is based on a brief screener and is subject to reporting error. The imaging samples are smaller and not fully representative of the original ABCD cohort. Finally, indirect associations were small and explain only a limited fraction of the diet–cognition covariance.

## Conclusions

A cognition-aligned dietary pattern was associated with crystallized and fluid cognition in held-out families, and the five unsupervised dietary PCs were jointly associated with both outcomes. BMI-adjusted brain analyses and complete all-ROI reporting identified bilateral fornix FA as the only structural class with FDR-corrected indirect associations in both cognitive domains. These cross-sectional results identify a selective pattern of shared covariance centered on WM memory pathways; they do not establish a causal nutritional mechanism.

## Supplementary Information

Below is the link to the electronic supplementary material.

Supplementary Material 1

## Data Availability

No datasets were generated or analysed during the current study.
