# Supplementary material for: Brain structural pathways linking dietary patterns and cognitive performance in adolescents
Source: Brain Struct Funct. 2026 Aug 29;231(7):129. doi: 10.1007/s00429-026-03190-w (PMC13525954; doi:10.1007/s00429-026-03190-w)
Supplement: Supplementary file 1 [file 429_2026_3190_MOESM1_ESM.pdf]

Supplementary material

1 Supplementary methods

1.1 Wave timing and cross-sectional interpretation

ABCD follow-up waves are scheduled relative to each participant's baseline visit rather than a common calendar date. Release 5.1 contains a partial four-year follow-up because releases are annual snapshots of a rolling cohort. Nutrition, cognition, and MRI measures used here were all drawn from the four-year wave and were treated as concurrent. Available assessment dates indicated that many measurements occurred on the same day, but there was no universal temporal ordering that would justify causal mediation. Figure S1 represents the monthly assessment-count.

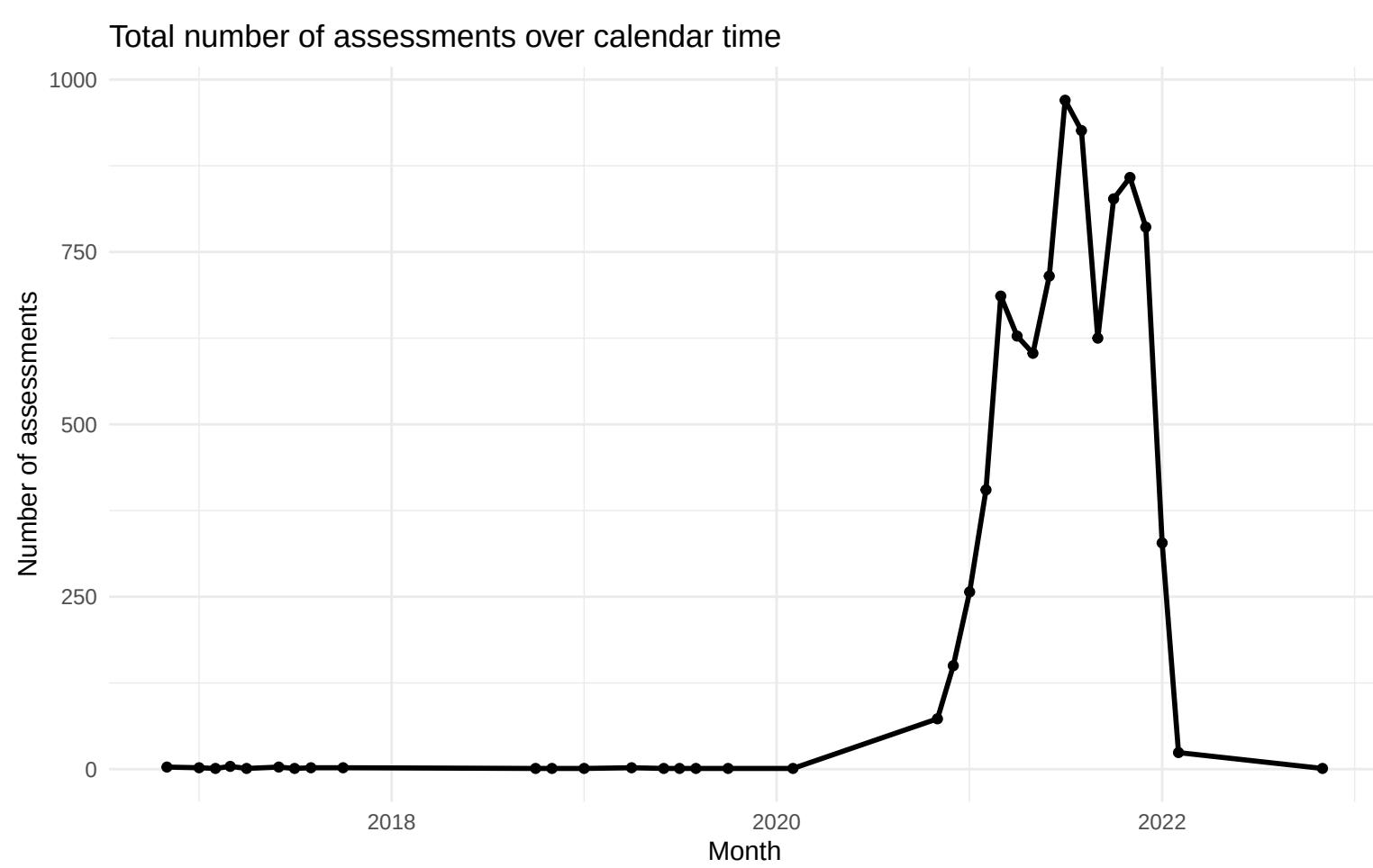

Figure S1: Calendar timing of four-year assessments in ABCD Release 5.1.

Most 4-year assessments in Release 5.1 were conducted during 2021, with a noticeable drop in December 2021 and early 2022. This decline may reflect disruptions associated with the rapid spread of the Omicron variant, though release-specific truncation and other administrative factors cannot be ruled out. Rolling enrollment and COVID disruptions made the realized calendar window broader than the nominal wave label suggests. This follows directly from the ABCD rolling design and COVID-era testing changes documented in the release materials (ABCD Study Annual Release 5.1, 2023).

Focusing on this release may limit, though not eliminate, the influence of longer-run COVID-related effects. Participants assessed earlier in the pandemic era were likely less

exposed to prolonged post-pandemic disruptions than those assessed later, which may be particularly relevant for nutrition and cognitive related measures. We therefore sought to minimize potential heterogeneity associated with later-pandemic conditions.

1.2 Sample structure, missingness, and anthropometric quality control

The main merged sample contained 3,308 adolescents. There were 2,340 singleton families and 479 multi-child families containing 968 children. Imaging sample sizes are summarized in Figure S2. Missingness by variable is shown in Figure S3.

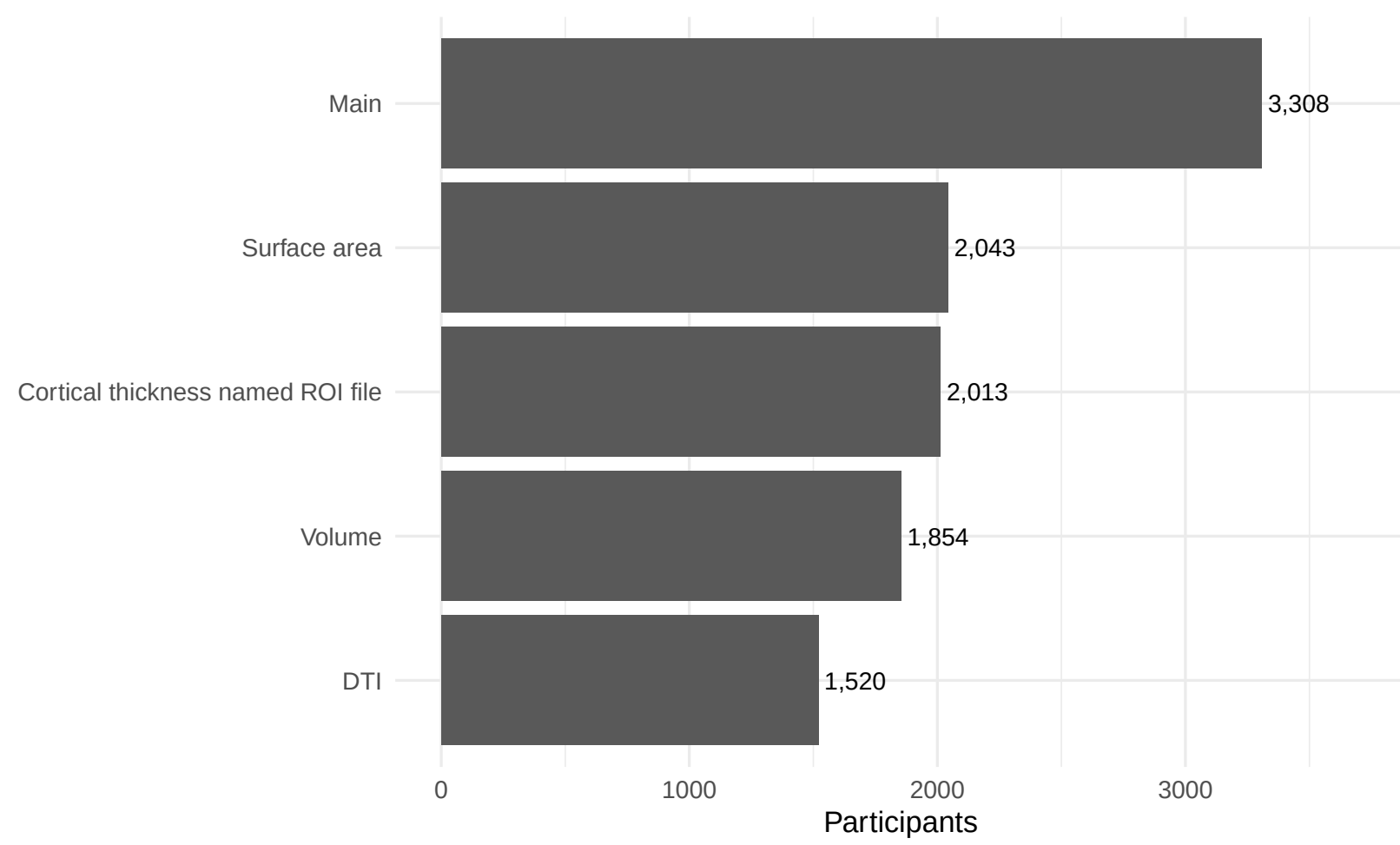

Figure S2: Available sample size by imaging modality.

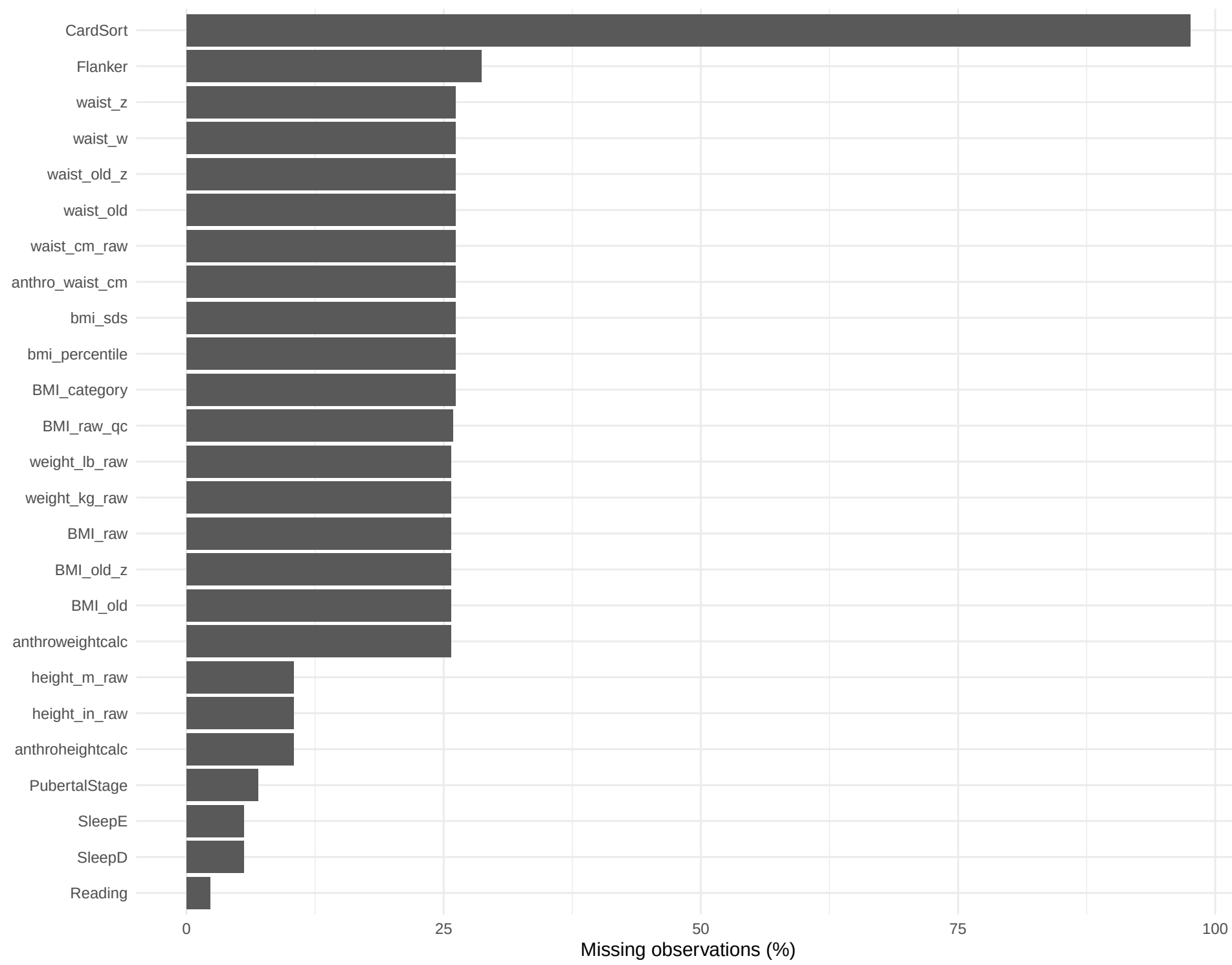

Figure S3: Variables with the highest percentages of missing observations.

BMI was calculated from measured height and weight. Height and weight were required to fall within prespecified plausible adolescent ranges before BMI SDS was calculated from CDC age- and sex-specific LMS parameters. Of 2,457 participants with complete height and weight, 2,451 passed quality control; BMI SDS was nonmissing for 2,444 participants after matching to the LMS table. Waist circumference was winsorized and standardized separately.

Category	N
Complete height and weight	2457
Passed anthropometric QC	2451
Failed anthropometric QC among complete records	6
Nonmissing BMI SDS after QC	2444

Table S1: Anthropometric quality-control summary for BMI-SDS analyses.

Statistic	Value
Children in singleton families	2340
Children in multi-child families	968
Singleton families	2340
Multi-child families	479

Table S2: Family structure in the analytic sample.

Analysis	N
Main	3308
DTI	1520
Surface area	2043
Cortical thickness named ROI file	2013
Volume	1854

Table S3: Sample sizes in the uploaded analytic datasets.

1.3 Descriptive Characteristics of the Analytic sample

Variable	Mean (SD)	Range
**Participant Characteristics (N=3,308)**		
Age, years	13.7 (0.72)	12.0–15.0
Female sex, n (%)	1,555 (47.0)	–
Pubertal stage	3.59 (0.74)	1.0–5.0
Income-to-needs ratio	3.87 (2.62)	0.06–14.35
Socioeconomic status (composite)	0.085 (0.87)	-3.72–1.69
Sleep duration, hours/night	6.7 (1.20)	2.71–11.0
Sleep efficiency, %	73.2 (16.4)	24.53–100.0
Social jetlag, hours	2.05 (1.59)	-11.28–11.54
**Anthropometric Measures**		
Body weight	135.67 (37.17)	77–266
Height	64.88 (3.43)	56–73
Body mass index	22.59 (5.6)	14.52–42.88
Waist circumference	31.75 (5.75)	23–55
**Dietary Intake (servings/week)**		
Fruits (excluding juice)	1.01 (0.78)	0.0–5.71
Vegetables (non-potato)	0.58 (0.44)	0.0–3.94
Whole grains	0.38 (0.39)	0.0–3.97
Dairy products	1.02 (0.79)	0.0–7.98
Meat, poultry, and fish	1.95 (1.76)	0.0–22.01
Legumes and nuts	0.05 (0.09)	0.0–0.96
Potatoes (including fries)	0.2 (0.19)	0.0–1.71
**Beverages and Condiments (daily intake)**		
Soft drinks, g/day	91.42 (133.42)	0.0–775.0
Sugar-sweetened beverages, kcal/day	37.73 (55.06)	0.0–319.8
Condiments (ketchup, salsa), g/day	1.58 (2.78)	0.0–15.5
**Macronutrients and Diet Quality**		
Total energy intake (standardized)^b	914.67 (477.83)	175–2603
Added sugars, tsp/day	5.63 (4.72)	0.0–56.32
Saturated fat, g/day	13.36 (8.17)	0.0–103.6
Fiber intake	8.17 (4.71)	0.98–24.28
Glycemic index	50.1 (4.34)	40.0–62.4
Glycemic load	50.26 (26.05)	9.2–138.81

Table S4: Participant Characteristics and Dietary Intake Patterns (standard deviation in parenthesis)

Notes: Income-to-needs ratio calculated based on 2021 federal poverty thresholds adjusted for household size. Socioeconomic status represents composite z-score incorporating parental education, income, and occupation. Missing data rates: Dietary (2.1%), Cognitive (4.8%), Covariates (3.2–12.1%).

Participants represented diverse socioeconomic backgrounds, with a mean income-to-needs ratio of 3.87 (sd=2.62) and were in mid-puberty on average (mean pubertal stage 3.59, sd=0.74).

1.4 Representativeness of the synthetic sample

The analytic subsamples differ from the full cohort primarily due to missing or excluded MRI scans; we therefore provide descriptive comparisons between included vs. excluded participants (i.e. those who have not yet been tested in release 5.1) in INR, race/ethnicity and sex. We were unable to formally compare age between included and excluded participants, because age at a visit is only observed for youth who attended that visit and completed the relevant assessments. However, the ABCD sampling frame and the relatively narrow age range at this wave reduce (but do not eliminate) concerns about major age-related selection biases.

Variable	Included (n = 3,308)	Excluded (n = 8,560)	Test statistic	p-value
Income-to-needs, m (sd)	3.87 (2.62)	3.56 (2.67)	$t(6477.7) = 5.56$, $d = 0.12$	$< .001$
Sex :				
% male / female / other	52.8 / 47.2 / 0.0	51.9 / 48.1 / 0.04	$\chi^2(2) = 1.96$	.38
Race/ethnicity :				
% non-Hispanic White	61.9	48.2		
non-Hispanic Black	8.5	17.6	χ^2 (sim.) $= 233.22$	$< .001$
Hispanic/Latino	18.3	21.1		
Asian	1.7	2.3		
Other / multiracial	9.5	10.9		

Table S5: Comparison of adolescents included in the analytic sample and those excluded from the present analyses.

Note: Values are percentages within inclusion group. Race/ethnicity categories follow the ABCD Study combined race/ethnicity variable (non-Hispanic White, non-Hispanic Black, Hispanic/Latino, non-Hispanic Asian, other/multiracial). The χ^2 test for race/ethnicity used a simulated p-value with $B = 10{,}000$ replicates.

The comparisons indicate that adolescents in the analytic sample had slightly higher income-to-needs ratios than those excluded (m = 3.87 vs. 3.56, t(6477.7) = 5.56, p < 0.001, Cohen's d = 0.12), whereas sex distributions were virtually identical across groups ($\chi^2(2) = 1.96$, p = 0.38). Race/ethnicity distributions differed significantly (χ^2, simulated p < 0.001), with the analytic sample including a higher proportion of non-Hispanic White adolescents and lower proportions of Black, Hispanic, and Asian adolescents compared with

the full ABCD cohort (see Table S2 for details). We acknowledge that this analytic sample is not fully representative of the ABCD cohort and that this may limit generalizability.

1.5 Exact cognitive-composite definitions and missing-data sensitivity

Crystallized cognition was calculated as the mean of standardized Oral Reading Recognition and Picture Vocabulary scores. Fluid cognition was calculated as the mean of standardized Flanker Inhibitory Control and Attention, List Sorting Working Memory, and Picture Sequence/Episodic Memory scores. These are study-specific composites assembled from NIH Toolbox tasks. Card Sort was not used because approximately 97.6% of observations were missing at the four-year wave. Predictive mean matching generated five completed datasets; the primary pipeline used the first completed dataset to reproduce the original score construction. Separate complete-case cross-fitted analyses were performed to assess sensitivity to cognitive imputation.

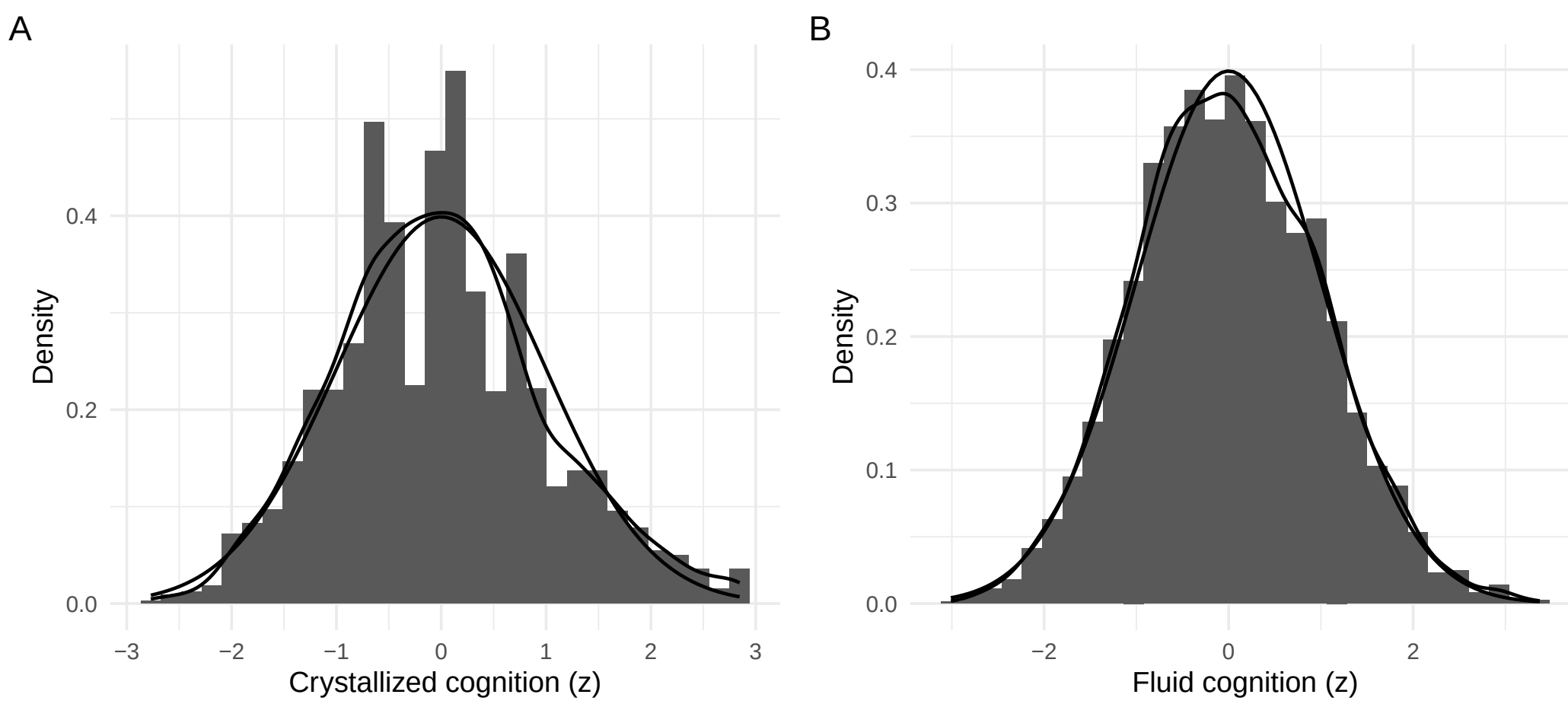

Figure S4: Distributions of the standardized crystallized and fluid cognitive composites.

1.6 Neuroimaging data and ROI definitions

We used ABCD-distributed derived imaging metrics. T1-weighted images were acquired at 1-mm isotropic resolution and processed with the ABCD structural pipeline, including FreeSurfer 6.0 cortical reconstruction and Desikan–Killiany parcellation. Diffusion acquisition used multiple b-values and 103 directions; ABCD-derived diffusion metrics incorporated motion and distortion correction and tract-specific FA summaries. The analysis did not register a new atlas or reconstruct tracts from raw images. ABCD inclusion and quality-control flags were applied before analysis.

The WM family contained bilateral fornix, bilateral hippocampal cingulum, bilateral inferior longitudinal fasciculus, and forceps major FA. GM families contained 12 cortical-thickness ROIs, 12 surface-area ROIs, and 30 regional-volume ROIs. Regional-volume models included intracranial volume, area models included total cortical area, and thickness models included mean cortical thickness. All listed ROIs were tested in the revised indirect-effect analysis; no negative-control ROI was used as a substitute for comprehensive reporting.

Region	Structure Type
Right Fornix	FA
Left Fornix	FA
Right Hippocampal Cingulum	FA
Left Hippocampal Cingulum	FA
Right Inferior Longitudinal Fasciculus	FA
Left Inferior Longitudinal Fasciculus	FA
Forceps Major	FA
Left Hippocampus	Volume
Right Hippocampus	Volume
Left Caudate	Volume
Right Caudate	Volume
Left Parahippocampal Gyrus	Volume
Right Parahippocampal Gyrus	Volume
Left Pars Opercularis	Volume
Right Pars Opercularis	Volume
Left Pars Orbitalis	Volume
Right Pars Orbitalis	Volume
Left Pars Triangularis	Volume
Right Pars Triangularis	Volume
Left Fusiform Gyrus	Volume
Right Fusiform Gyrus	Volume
Left Middle Temporal Gyrus	Volume
Right Middle Temporal Gyrus	Volume
Left Rostral Middle Frontal Gyrus	Volume
Right Rostral Middle Frontal Gyrus	Volume
Left Caudal Middle Frontal Gyrus	Volume
Right Caudal Middle Frontal Gyrus	Volume
Left Frontal Pole	Volume
Right Frontal Pole	Volume

Region	Structure Type
Left Insula	Volume
Right Insula	Volume
Left Precuneus	Volume
Right Precuneus	Volume
Left Postcentral Gyrus	Volume
Right Postcentral Gyrus	Volume
Left Paracentral Lobule	Volume
Right Paracentral Lobule	Volume
Left Parahippocampal Gyrus	Thickness
Right Parahippocampal Gyrus	Thickness
Left Pars Opercularis	Thickness
Right Pars Opercularis	Thickness
Left Pars Orbitalis	Thickness
Right Pars Orbitalis	Thickness
Left Pars Triangularis	Thickness
Right Pars Triangularis	Thickness
Left Rostral Middle Frontal Gyrus	Thickness
Right Rostral Middle Frontal Gyrus	Thickness
Left Caudal Middle Frontal Gyrus	Thickness
Right Caudal Middle Frontal Gyrus	Thickness
Left Dorsolateral Prefrontal Cortex	Area
Right Dorsolateral Prefrontal Cortex	Area
Left Parahippocampal Gyrus	Area
Right Parahippocampal Gyrus	Area
Left Entorhinal Cortex	Area
Right Entorhinal Cortex	Area
Left Pars Opercularis	Area
Right Pars Opercularis	Area
Left Temporal Pole	Area
Right Temporal Pole	Area
Left Rostral Middle Frontal	Area
Right Rostral Middle Frontal	Area

Table S6: Brain and tracts retained from the ABCD derived measures

1.7 Covariate specifications, family structure, and multiple testing

The primary specification included age, sex, pubertal stage, SES, INR, sleep duration, sleep efficiency, social jetlag, BMI SDS, and site for imaging models. Ethnicity was excluded from the primary models and was added only in a sensitivity specification. Additional models omitted adiposity or included BMI SDS plus waist circumference. Cognition models used family random intercepts. The complete-case FA sample had one participant per family, so a family random intercept was not estimable; other modality models and

the bootstrap resampling respected family clusters.

Benjamini–Hochberg FDR correction was applied within each modality, cognitive outcome, and effect family. For the PC-based FA analysis, correction additionally covered all focal-PC-by-tract tests. All indirect-effect models are reported below, not only those reaching nominal or corrected significance.

2 Supplementary results

2.1 Dietary preprocessing and PCA

The food PCA used ten Block Kids Food Screener variables. Variables were winsorized at the first and 99th percentiles, transformed with $\log(1 + x)$ where appropriate, and standardized. PCA was applied to the correlation matrix. Five components were retained because they provided an interpretable representation of dietary patterns and together explained 77.0% of variance. The scree plot indicates a strong first component and a secondary elbow near PC2–PC3; the additional PCs preserve smaller but interpretable contrasts for downstream multivariate analysis.

PC	Eigenvalue	Proportion	Cumulative
PC1	3.1288	0.3129	0.3129
PC2	1.9398	0.1940	0.5069
PC3	0.9279	0.0928	0.5996
PC4	0.8466	0.0847	0.6843
PC5	0.8295	0.0829	0.7673
PC6	0.7695	0.0770	0.8442
PC7	0.6156	0.0616	0.9058
PC8	0.5103	0.0510	0.9568
PC9	0.4311	0.0431	0.9999
PC10	0.0011	0.0001	1.0000

Table S7: Variance explained by the food principal components.

Food Category	**PC1**	**PC2**	**PC3**	**PC4**	**PC5**
Fruit (excluding juice)	0.2904	0.1281	-0.4190	-0.0297	-0.7375
Vegetable (non potato)	0.4121	0.2252	0.0131	0.1166	-0.2022
Potato	0.3625	-0.0093	0.2555	-0.2274	-0.0132
Whole grains	0.2698	0.2274	-0.5732	-0.0431	0.3305
Meat/Poultry/Fish	0.4187	0.0580	0.1819	-0.0644	0.0827
Dairy	0.3478	0.1197	-0.2024	0.1011	0.5448
Legumes	0.2688	0.1053	0.4314	0.7133	-0.0470
Sugary beverages	0.2221	-0.6526	-0.1080	0.0614	0.0016
Soft drinks	0.2210	-0.6525	-0.1097	0.0647	-0.0008
Ketchup and salsa	0.2737	0.0251	0.3828	-0.6331	0.0054
**Component Interpretations**					
PC1 Broad Food Consumption					
PC2 Low Sugary-Beverage Intake					
PC3 Food-Pattern Contrast					
PC4 Legume–Condiment Contrast					
PC5 Dairy/Whole-Grain–Fruit Contrast					

Table S8: Loadings for the first five food principal components.

The first component, explaining 31.3% of dietary variance, reflected a broad dimension of food intake, with positive loadings across most food groups and the largest loadings for meat/poultry/fish, vegetables, potatoes, and dairy. The second component, explaining 19.4%, was dominated by low consumption of sugary beverages and soft drinks. Subsequent components represented more specific dietary contrasts and were therefore interpreted primarily from their loading profiles rather than assigned strong a priori dietary-quality labels.

2.2 PolyIQ construction and family-level cross-fitting

PolyIQ is an outcome-specific weighted sum of the five dietary PCs. The weights were estimated with elastic net ($\alpha = 0.5$), with λ selected by cross-validation. Ordinary-regression weights produced nearly identical scores, demonstrating that the results are not dependent on penalization. The supervised score is not a normative diet-quality index.

For downstream inference, families were divided into five outer folds. In each outer iteration, elastic-net weights were estimated in the remaining families using inner family-level cross-validation. Those weights were applied to the held-out families. Held-out scores were standardized and averaged across ten repeated partitions. A participant's own cognition and the cognition of family members therefore did not determine the score used for that participant.

Component	Outcome	Weight
PC1	Crystallized	0.0607
PC2	Crystallized	0.0799
PC3	Crystallized	-0.0561
PC4	Crystallized	0.0734
PC5	Crystallized	0.0379
PC1	Fluid	0.0453
PC2	Fluid	0.0258
PC3	Fluid	-0.0286
PC4	Fluid	0.0378
PC5	Fluid	0.0289

Table S9: Elastic-net weights used to construct the original in-sample PolyIQ scores.

Outcome	Correlation	MeanDifference_OLS_minus_EN	SDDifference
Crystallized	1.0000	0.0000	0.0012
Fluid	1.0000	0.0000	0.0007

Table S10: Comparison of PolyIQ scores constructed by elastic net and ordinary regression.

2.3 Cross-fitting diagnostics, cognition models, and PC benchmarks

Cross-fitted and in-sample scores were nearly identical, but the cross-fitted associations with cognition were modestly smaller. This is the expected pattern when own-outcome reuse is removed. Primary estimates remained significant with BMI SDS, with BMI SDS plus waist, and when ethnicity was included. Complete-case cognitive analyses were also similar.

Outcome	r(InSampleScore,Outcome)	r(CrossFitScore,Outcome)	r(InSample,CrossFit)
Crystallized	0.2066	0.1999	0.9996
Fluid	0.1451	0.1331	0.9989

Table S11: Comparison of the original in-sample and family-level cross-fitted PolyIQ scores.

Specification	Outcome	Exposure	N	estimate	se	statistic	p_value
Primary: BMI SDS, no ethnicity	crystIQ	PolyIQ_cryst_cf	2145	0.0994	0.0178	5.5830	0.0000
No adiposity, no ethnicity	crystIQ	PolyIQ_cryst_cf	2905	0.0886	0.0154	5.7738	0.0000
BMI SDS + waist, no ethnicity	crystIQ	PolyIQ_cryst_cf	2128	0.0979	0.0179	5.4725	0.0000
BMI SDS + ethnicity	crystIQ	PolyIQ_cryst_cf	2145	0.0814	0.0176	4.6212	0.0000
Primary: BMI SDS, no ethnicity	fluidIQ	PolyIQ_fluid_cf	2145	0.0636	0.0151	4.2121	0.0000
No adiposity, no ethnicity	fluidIQ	PolyIQ_fluid_cf	2905	0.0550	0.0133	4.1252	0.0000
BMI SDS + waist, no ethnicity	fluidIQ	PolyIQ_fluid_cf	2128	0.0632	0.0151	4.1753	0.0000
BMI SDS + ethnicity	fluidIQ	PolyIQ_fluid_cf	2145	0.0509	0.0151	3.3762	0.0007

Table S12: Mixed-effects models using family-level cross-fitted PolyIQ scores (normalized).

Outcome	N	estimate	se	statistic	p_value
Crystallized complete case	2087	0.1023	0.0180	5.6799	0.0000
Fluid complete case	1980	0.0718	0.0157	4.5638	0.0000

Table S13: Complete-case cognitive sensitivity analyses using cross-fitted PolyIQ.

Model	χ^2	df	p-value	N
Cryst_Primary	36.5763	5.0000	0.0000	2145
Fluid_Primary	27.3138	5.0000	0.0000	2145
Cryst_Ethnicity	25.2217	5.0000	0.0001	2145
Fluid_Ethnicity	19.6413	5.0000	0.0015	2145

Table S14: Likelihood-ratio tests of the joint contribution of all five dietary principal components.

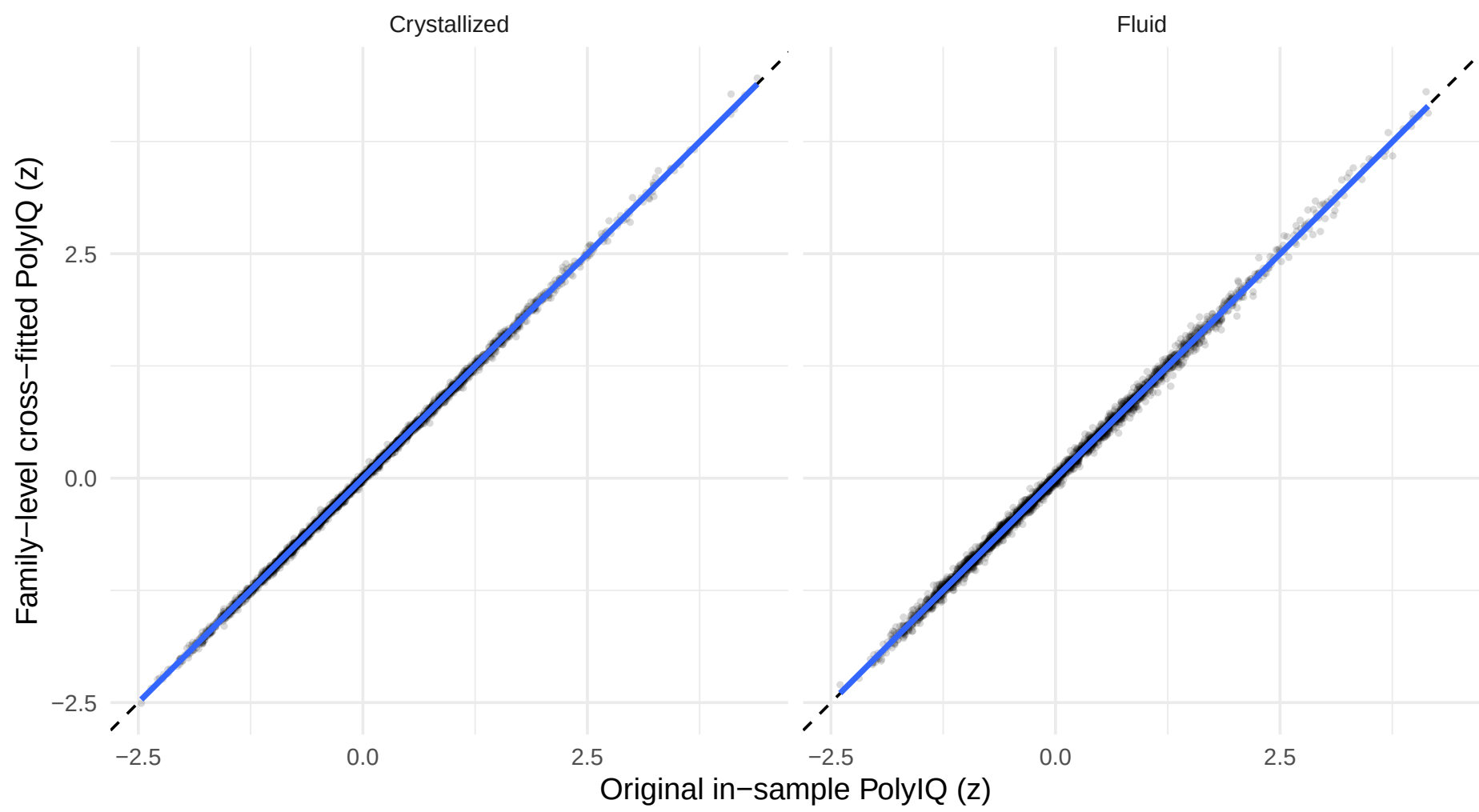

Figure S5: Comparison of original in-sample and family-level cross-fitted PolyIQ scores.

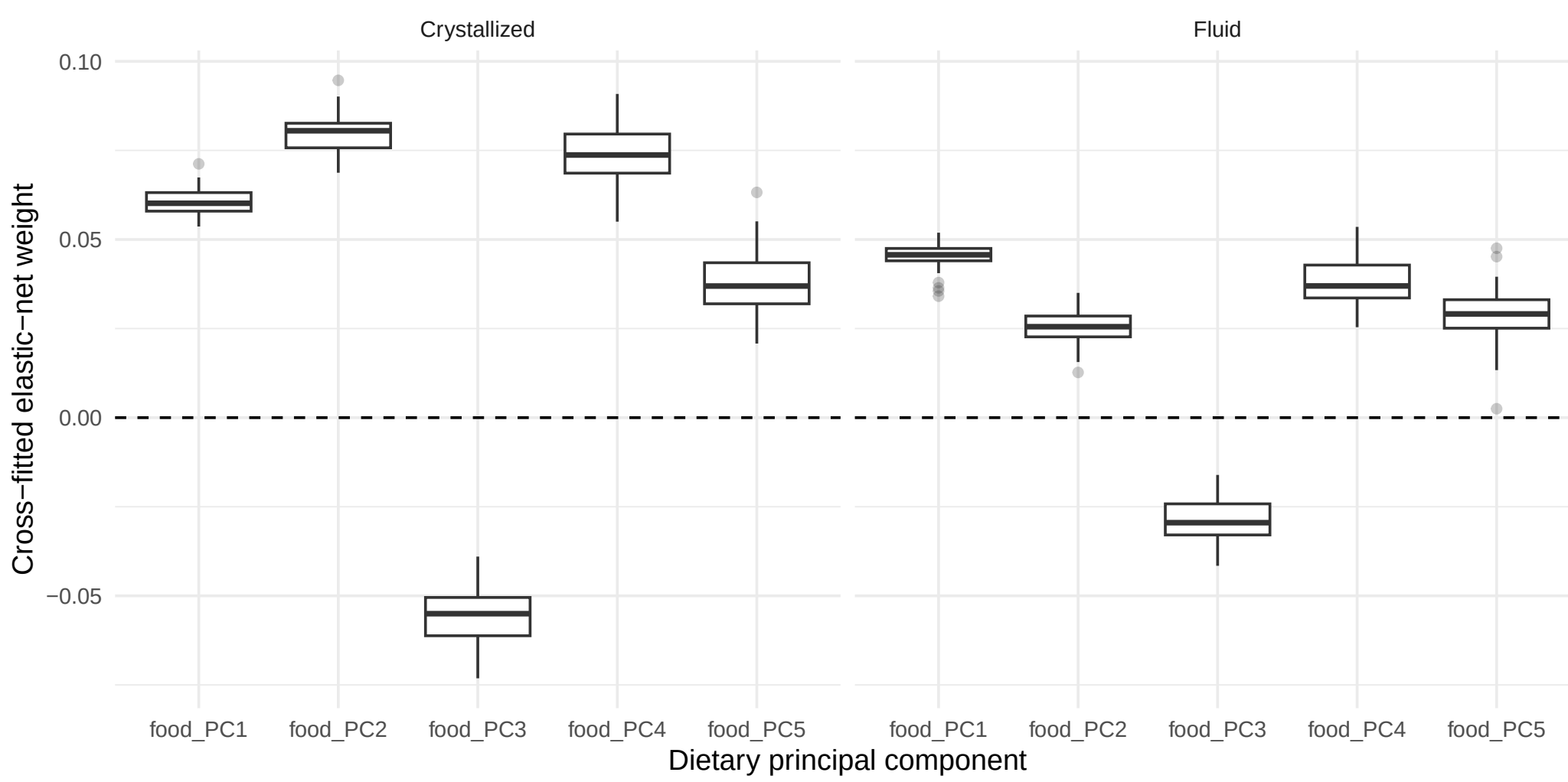

Figure S6: Stability of elastic-net PC weights across repeated outer folds.

2.4 Brain–cognition associations across adiposity and ethnicity specifications

Figure S7 shows the FA coefficients across the four prespecified specifications. Bilateral fornix associations were stable across all specifications. BMI adjustment did not attenuate

	Crystalized	Fluid	Crystalized	Fluid
(Intercept)	0.057	0.009	0.135***	0.058*
	(0.031)	(0.026)	(0.034)	(0.028)
PC1	0.036***	0.035***	0.030**	0.031***
	(0.010)	(0.008)	(0.010)	(0.008)
PC2	0.042***	0.006	0.037**	0.003
	(0.013)	(0.011)	(0.012)	(0.011)
PC3	−0.028	−0.012	−0.017	−0.005
	(0.018)	(0.015)	(0.018)	(0.015)
PC4	0.042*	0.019	0.036*	0.015
	(0.018)	(0.015)	(0.018)	(0.015)
PC5	0.050**	0.048**	0.036	0.040*
	(0.019)	(0.016)	(0.019)	(0.016)
SJL_z	−0.161***	−0.077***	−0.143***	−0.065***
	(0.022)	(0.019)	(0.022)	(0.019)
SleepD_z	0.007	0.020	0.016	0.026
	(0.033)	(0.028)	(0.033)	(0.028)
SleepE_z	0.008	0.009	0.008	0.008
	(0.033)	(0.028)	(0.032)	(0.027)
age_z	−0.181**	−0.078	−0.167**	−0.066
	(0.056)	(0.046)	(0.055)	(0.046)
PubertalStage_z	0.068**	−0.007	0.067**	−0.008
	(0.023)	(0.019)	(0.022)	(0.019)
Sex Female	−0.046	−0.005	−0.043	−0.001
	(0.046)	(0.038)	(0.045)	(0.038)
SES_z	0.141***	0.122***	0.108***	0.100***
	(0.023)	(0.019)	(0.023)	(0.019)
INR_z	0.146***	0.077***	0.116***	0.059***
	(0.021)	(0.018)	(0.021)	(0.018)
bmi_sds	−0.034*	−0.011	−0.021	−0.003
	(0.017)	(0.014)	(0.016)	(0.014)
EthnRace2			−0.618***	−0.399***
			(0.075)	(0.062)
EthnRace3			−0.226***	−0.130**
			(0.051)	(0.042)
EthnRace4			0.216	0.132
			(0.128)	(0.107)
EthnRace5			−0.020	−0.031
			(0.060)	(0.050)
AIC	5056.669	4327.772	4985.335	4289.877
BIC	5153.074	4424.177	5104.424	4408.966
Log Likelihood	−2511.335	−2146.886	−2471.668	−2123.939
Num. obs.	2145	2145	2145	2145
Num. groups: famID	1864	1864	1864	1864
Var: famID (Intercept)	0.332	0.144	0.308	0.131
Var: Residual	0.303	0.296	0.302	0.299

$^{***}p < 0.001$; $^{**}p < 0.01$; $^{*}p < 0.05$

Table S15: Cognition models including all five food principal components.

the primary FA findings. Left hippocampal cingulum FA for fluid cognition was sensitive to inclusion of ethnicity, but the bilateral fornix findings and the right cingulum findings remained.

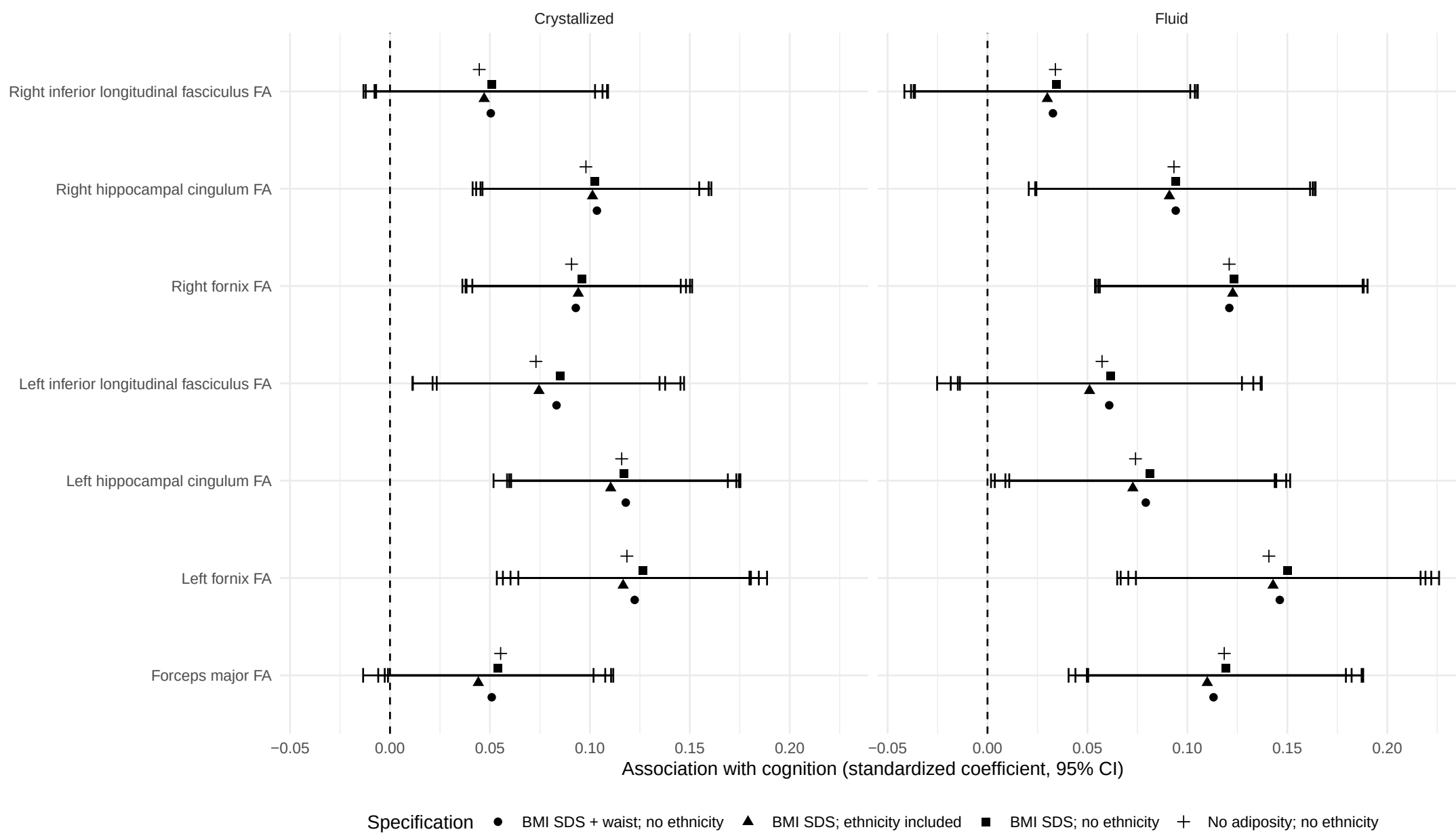

Figure S7: FA–cognition associations across adiposity and ethnicity specifications. Points are coefficients and bars are 95% confidence intervals.

In the BMI-SDS/no-ethnicity primary model, all four fornix outcome combinations survived FDR correction. Both hippocampal cingulum tracts were associated with crystallized cognition; both were also associated with fluid cognition in the primary model. Left inferior longitudinal fasciculus FA was associated with crystallized cognition, and forceps major FA with fluid cognition. Regional-volume associations surviving correction were observed only for crystallized cognition in the primary specification. Surface-area and thickness results did not survive correction.

2.5 Complete all-ROI indirect-effect results

Figure S8 plots all indirect effects and corrected confidence intervals. Table S16 reports every tested indirect-effect model, including nonsignificant models, as requested by Reviewer 5.

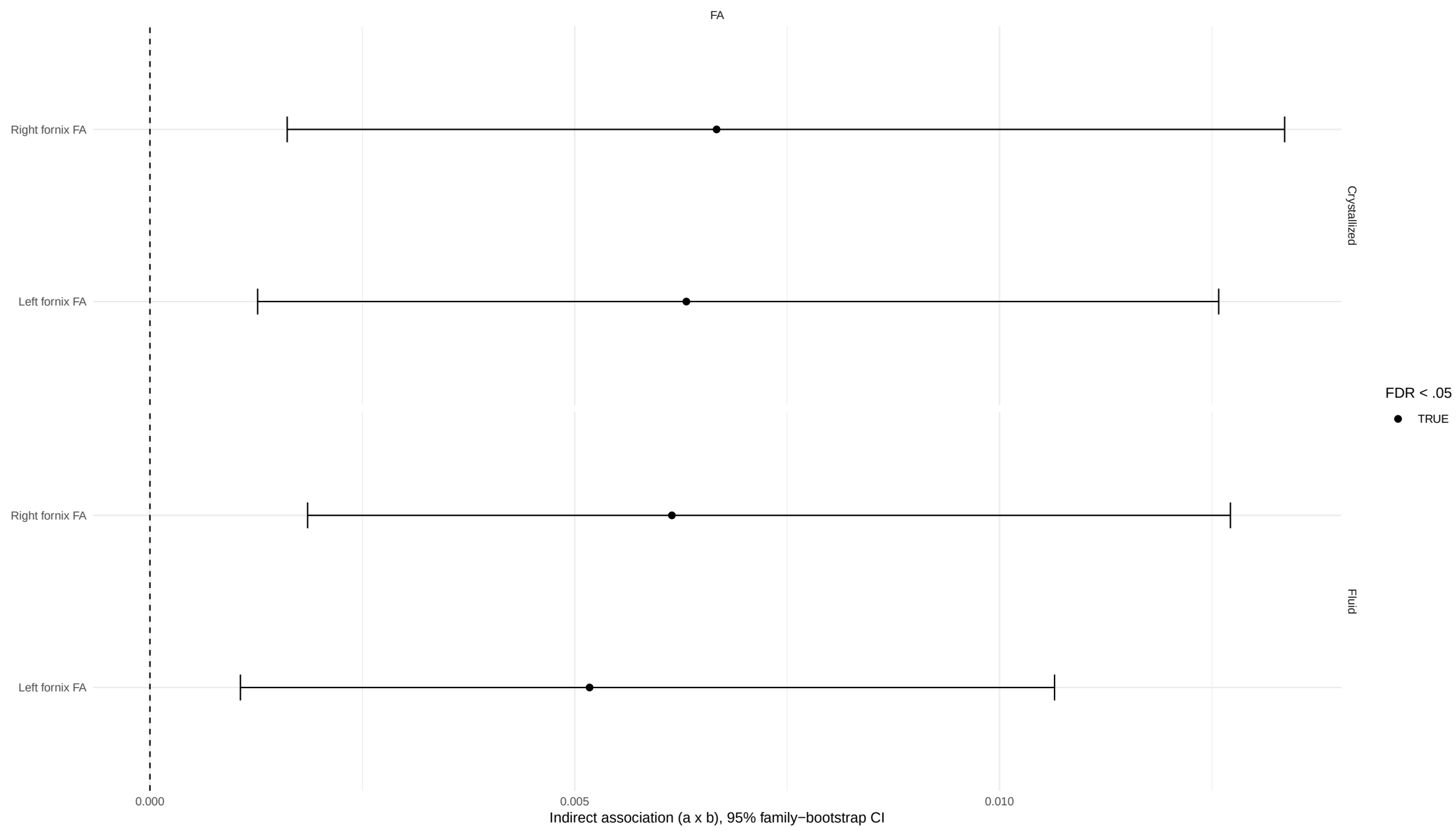

Figure S8: All prespecified indirect-effect estimates using cross-fitted PolyIQ.

Table S16: All tested cross-fitted indirect-effect models

Modality	Outcome	Mediator	N	Estimate	95% CI	P	P_{FDR}
FA	Crystallized	Right fornix	1423	0.0067	[0.0016, 0.0134]	0.004	0.028
FA	Crystallized	Left fornix	1423	0.0063	[0.0013, 0.0126]	0.012	0.042
FA	Crystallized	Left inferior longitudinal fasciculus	1423	0.0029	[-0.0001, 0.0073]	0.072	0.168
FA	Crystallized	Left hippocampal cingulum	1423	0.0036	[-0.0014, 0.0098]	0.174	0.304
FA	Crystallized	Forceps major	1423	0.0013	[-0.0011, 0.0045]	0.354	0.396
FA	Crystallized	Right inferior longitudinal fasciculus	1423	0.0010	[-0.0011, 0.0044]	0.358	0.396
FA	Crystallized	Right hippocampal cingulum	1423	0.0020	[-0.0024, 0.0069]	0.396	0.396
FA	Fluid	Right fornix	1423	0.0061	[0.0019, 0.0127]	< 0.001	< 0.001
FA	Fluid	Left fornix	1423	0.0052	[0.0011, 0.0106]	0.012	0.042
FA	Fluid	Left inferior longitudinal fasciculus	1423	0.0011	[-0.0006, 0.0040]	0.220	0.465
FA	Fluid	Left hippocampal cingulum	1423	0.0013	[-0.0008, 0.0048]	0.300	0.465
FA	Fluid	Forceps major	1423	0.0018	[-0.0015, 0.0063]	0.332	0.465
FA	Fluid	Right hippocampal cingulum	1423	0.0011	[-0.0016, 0.0048]	0.402	0.469
FA	Fluid	Right inferior longitudinal fasciculus	1423	0.0004	[-0.0009, 0.0025]	0.702	0.702
Surface area	Crystallized	Left pars opercularis	1774	-0.0005	[-0.0029, 0.0014]	0.572	0.956
Surface area	Crystallized	Right parahippocampal gyrus	1774	0.0002	[-0.0011, 0.0017]	0.768	0.956
Surface area	Crystallized	Right rostral middle frontal region	1774	-0.0002	[-0.0017, 0.0011]	0.772	0.956
Surface area	Crystallized	Right temporal pole	1774	0.0002	[-0.0010, 0.0018]	0.778	0.956
Surface area	Crystallized	Left entorhinal cortex	1774	0.0002	[-0.0012, 0.0024]	0.798	0.956
Surface area	Crystallized	Left parahippocampal gyrus	1774	-0.0002	[-0.0016, 0.0012]	0.816	0.956
Surface area	Crystallized	Right dorsolateral prefrontal cortex	1774	0.0002	[-0.0010, 0.0017]	0.840	0.956
Surface area	Crystallized	Left rostral middle frontal region	1774	-0.0002	[-0.0021, 0.0018]	0.868	0.956
Surface area	Crystallized	Left temporal pole	1774	0.0001	[-0.0015, 0.0020]	0.882	0.956
Surface area	Crystallized	Left dorsolateral prefrontal cortex	1774	-0.0001	[-0.0015, 0.0009]	0.884	0.956
Surface area	Crystallized	Right entorhinal cortex	1774	0.0000	[-0.0010, 0.0011]	0.928	0.956
Surface area	Crystallized	Right pars opercularis	1774	0.0000	[-0.0016, 0.0016]	0.956	0.956
Surface area	Fluid	Left pars opercularis	1774	-0.0009	[-0.0032, 0.0005]	0.242	0.980
Surface area	Fluid	Left rostral middle frontal region	1774	-0.0009	[-0.0030, 0.0008]	0.354	0.980
Surface area	Fluid	Left dorsolateral prefrontal cortex	1774	-0.0003	[-0.0018, 0.0009]	0.674	0.980
Surface area	Fluid	Left temporal pole	1774	0.0004	[-0.0015, 0.0026]	0.688	0.980
Surface area	Fluid	Right parahippocampal gyrus	1774	0.0002	[-0.0009, 0.0019]	0.720	0.980
Surface area	Fluid	Right rostral middle frontal region	1774	-0.0003	[-0.0017, 0.0010]	0.774	0.980
Surface area	Fluid	Left entorhinal cortex	1774	-0.0002	[-0.0014, 0.0010]	0.810	0.980
Surface area	Fluid	Left parahippocampal gyrus	1774	-0.0001	[-0.0014, 0.0010]	0.888	0.980
Surface area	Fluid	Right entorhinal cortex	1774	0.0000	[-0.0008, 0.0009]	0.902	0.980
Surface area	Fluid	Right temporal pole	1774	-0.0000	[-0.0014, 0.0013]	0.908	0.980
Surface area	Fluid	Right dorsolateral prefrontal cortex	1774	0.0000	[-0.0012, 0.0013]	0.952	0.980
Surface area	Fluid	Right pars opercularis	1774	0.0000	[-0.0017, 0.0015]	0.980	0.980
Cortical thickness	Crystallized	Right rostral middle frontal gyrus	1743	-0.0016	[-0.0053, 0.0004]	0.146	1.000
Cortical thickness	Crystallized	Left pars orbitalis	1743	0.0009	[-0.0008, 0.0035]	0.416	1.000
Cortical thickness	Crystallized	Left pars triangularis	1743	0.0006	[-0.0008, 0.0030]	0.458	1.000
Cortical thickness	Crystallized	Left rostral middle frontal gyrus	1743	0.0006	[-0.0014, 0.0031]	0.526	1.000
Cortical thickness	Crystallized	Right caudal middle frontal gyrus	1743	0.0005	[-0.0010, 0.0026]	0.540	1.000
Cortical thickness	Crystallized	Left pars opercularis	1743	-0.0002	[-0.0022, 0.0012]	0.826	1.000
Cortical thickness	Crystallized	Right parahippocampal gyrus	1743	-0.0001	[-0.0012, 0.0012]	0.876	1.000
Cortical thickness	Crystallized	Right pars orbitalis	1743	0.0001	[-0.0012, 0.0012]	0.910	1.000
Cortical thickness	Crystallized	Left caudal middle frontal gyrus	1743	0.0001	[-0.0013, 0.0014]	0.942	1.000
Cortical thickness	Crystallized	Right pars opercularis	1743	-0.0000	[-0.0020, 0.0019]	0.948	1.000
Cortical thickness	Crystallized	Right pars triangularis	1743	0.0000	[-0.0013, 0.0013]	0.976	1.000
Cortical thickness	Crystallized	Left parahippocampal gyrus	1743	0.0000	[-0.0027, 0.0024]	1.000	1.000

Continued on next page

Table S16 – continued

Modality	Outcome	Mediator	N	Estimate	95% CI	P	P_{FDR}
Cortical thickness	Fluid	Right rostral middle frontal gyrus	1743	-0.0007	[-0.0030, 0.0006]	0.340	0.998
Cortical thickness	Fluid	Left pars orbitalis	1743	0.0009	[-0.0009, 0.0034]	0.352	0.998
Cortical thickness	Fluid	Right pars opercularis	1743	0.0008	[-0.0009, 0.0030]	0.358	0.998
Cortical thickness	Fluid	Left rostral middle frontal gyrus	1743	0.0005	[-0.0007, 0.0022]	0.510	0.998
Cortical thickness	Fluid	Right pars orbitalis	1743	0.0001	[-0.0009, 0.0013]	0.780	0.998
Cortical thickness	Fluid	Right caudal middle frontal gyrus	1743	0.0000	[-0.0008, 0.0011]	0.888	0.998
Cortical thickness	Fluid	Left pars opercularis	1743	0.0001	[-0.0012, 0.0016]	0.896	0.998
Cortical thickness	Fluid	Left pars triangularis	1743	0.0001	[-0.0010, 0.0014]	0.916	0.998
Cortical thickness	Fluid	Left parahippocampal gyrus	1743	-0.0000	[-0.0009, 0.0012]	0.934	0.998
Cortical thickness	Fluid	Right pars triangularis	1743	0.0000	[-0.0009, 0.0009]	0.946	0.998
Cortical thickness	Fluid	Right parahippocampal gyrus	1743	-0.0001	[-0.0014, 0.0014]	0.994	0.998
Cortical thickness	Fluid	Left caudal middle frontal gyrus	1743	-0.0000	[-0.0011, 0.0009]	0.998	0.998
Regional volume	Crystallized	Left parahippocampal gyrus	1611	0.0039	[0.0003, 0.0091]	0.026	0.780
Regional volume	Crystallized	Right middle temporal gyrus	1611	0.0028	[-0.0014, 0.0077]	0.176	0.996
Regional volume	Crystallized	Left pars opercularis	1611	0.0013	[-0.0005, 0.0048]	0.208	0.996
Regional volume	Crystallized	Right fusiform gyrus	1611	0.0015	[-0.0012, 0.0051]	0.316	0.996
Regional volume	Crystallized	Right parahippocampal gyrus	1611	0.0020	[-0.0025, 0.0064]	0.348	0.996
Regional volume	Crystallized	Left middle temporal gyrus	1611	0.0010	[-0.0014, 0.0044]	0.384	0.996
Regional volume	Crystallized	Right pars opercularis	1611	0.0007	[-0.0012, 0.0036]	0.478	0.996
Regional volume	Crystallized	Right insula	1611	-0.0007	[-0.0035, 0.0013]	0.482	0.996
Regional volume	Crystallized	Right pars orbitalis	1611	0.0007	[-0.0010, 0.0033]	0.514	0.996
Regional volume	Crystallized	Right pars triangularis	1611	0.0005	[-0.0010, 0.0028]	0.556	0.996
Regional volume	Crystallized	Right postcentral gyrus	1611	-0.0006	[-0.0029, 0.0014]	0.566	0.996
Regional volume	Crystallized	Left pars orbitalis	1611	0.0008	[-0.0023, 0.0049]	0.612	0.996
Regional volume	Crystallized	Right precuneus	1611	0.0009	[-0.0027, 0.0053]	0.614	0.996
Regional volume	Crystallized	Left precuneus	1611	0.0004	[-0.0018, 0.0025]	0.716	0.996
Regional volume	Crystallized	Right frontal pole	1611	-0.0003	[-0.0020, 0.0010]	0.736	0.996
Regional volume	Crystallized	Left rostral middle frontal gyrus	1611	-0.0005	[-0.0037, 0.0023]	0.742	0.996
Regional volume	Crystallized	Right paracentral lobule	1611	-0.0002	[-0.0026, 0.0015]	0.762	0.996
Regional volume	Crystallized	Right rostral middle frontal gyrus	1611	0.0002	[-0.0014, 0.0022]	0.780	0.996
Regional volume	Crystallized	Right caudate	1611	0.0002	[-0.0013, 0.0017]	0.876	0.996
Regional volume	Crystallized	Right caudal middle frontal gyrus	1611	-0.0001	[-0.0017, 0.0010]	0.882	0.996
Regional volume	Crystallized	Left insula	1611	-0.0001	[-0.0024, 0.0022]	0.910	0.996
Regional volume	Crystallized	Right hippocampus	1611	-0.0000	[-0.0016, 0.0014]	0.942	0.996
Regional volume	Crystallized	Left hippocampus	1611	-0.0000	[-0.0015, 0.0011]	0.958	0.996
Regional volume	Crystallized	Left frontal pole	1611	0.0001	[-0.0012, 0.0012]	0.966	0.996
Regional volume	Crystallized	Left pars triangularis	1611	-0.0001	[-0.0018, 0.0018]	0.968	0.996
Regional volume	Crystallized	Left caudate	1611	0.0000	[-0.0016, 0.0018]	0.984	0.996
Regional volume	Crystallized	Left postcentral gyrus	1611	0.0000	[-0.0012, 0.0012]	0.986	0.996
Regional volume	Crystallized	Left caudal middle frontal gyrus	1611	0.0000	[-0.0013, 0.0012]	0.994	0.996
Regional volume	Crystallized	Left fusiform gyrus	1611	0.0002	[-0.0040, 0.0042]	0.996	0.996
Regional volume	Crystallized	Left paracentral lobule	1611	-0.0000	[-0.0017, 0.0017]	0.996	0.996
Regional volume	Fluid	Left parahippocampal gyrus	1611	0.0017	[-0.0004, 0.0049]	0.104	0.994
Regional volume	Fluid	Left pars opercularis	1611	0.0008	[-0.0009, 0.0033]	0.378	0.994
Regional volume	Fluid	Right hippocampus	1611	0.0006	[-0.0005, 0.0025]	0.402	0.994
Regional volume	Fluid	Left paracentral lobule	1611	0.0007	[-0.0008, 0.0028]	0.436	0.994
Regional volume	Fluid	Right precuneus	1611	0.0007	[-0.0006, 0.0029]	0.436	0.994
Regional volume	Fluid	Right frontal pole	1611	-0.0004	[-0.0022, 0.0008]	0.540	0.994
Regional volume	Fluid	Left middle temporal gyrus	1611	0.0004	[-0.0008, 0.0020]	0.594	0.994
Regional volume	Fluid	Left rostral middle frontal gyrus	1611	-0.0003	[-0.0022, 0.0008]	0.594	0.994
Regional volume	Fluid	Right postcentral gyrus	1611	-0.0004	[-0.0023, 0.0010]	0.630	0.994
Regional volume	Fluid	Left insula	1611	0.0004	[-0.0014, 0.0028]	0.644	0.994
Regional volume	Fluid	Right caudate	1611	0.0004	[-0.0010, 0.0020]	0.678	0.994
Regional volume	Fluid	Left pars triangularis	1611	0.0003	[-0.0009, 0.0018]	0.682	0.994
Regional volume	Fluid	Left caudate	1611	0.0003	[-0.0014, 0.0026]	0.684	0.994
Regional volume	Fluid	Right fusiform gyrus	1611	0.0003	[-0.0009, 0.0021]	0.708	0.994

Continued on next page

Table S16 – continued

Modality	Outcome	Mediator	N	Estimate	95% CI	P	P_{FDR}
Regional volume	Fluid	Left precuneus	1611	0.0002	[-0.0011, 0.0021]	0.718	0.994
Regional volume	Fluid	Right paracentral lobule	1611	0.0002	[-0.0012, 0.0019]	0.746	0.994
Regional volume	Fluid	Right insula	1611	-0.0003	[-0.0027, 0.0020]	0.780	0.994
Regional volume	Fluid	Right pars opercularis	1611	0.0002	[-0.0009, 0.0020]	0.794	0.994
Regional volume	Fluid	Right caudal middle frontal gyrus	1611	-0.0001	[-0.0025, 0.0020]	0.860	0.994
Regional volume	Fluid	Right parahippocampal gyrus	1611	0.0001	[-0.0013, 0.0017]	0.862	0.994
Regional volume	Fluid	Right pars triangularis	1611	-0.0002	[-0.0014, 0.0012]	0.880	0.994
Regional volume	Fluid	Left hippocampus	1611	0.0001	[-0.0012, 0.0017]	0.906	0.994
Regional volume	Fluid	Right middle temporal gyrus	1611	0.0001	[-0.0012, 0.0015]	0.920	0.994
Regional volume	Fluid	Left caudal middle frontal gyrus	1611	0.0000	[-0.0012, 0.0014]	0.926	0.994
Regional volume	Fluid	Left postcentral gyrus	1611	0.0001	[-0.0012, 0.0014]	0.928	0.994
Regional volume	Fluid	Left frontal pole	1611	-0.0000	[-0.0014, 0.0011]	0.948	0.994
Regional volume	Fluid	Left pars orbitalis	1611	-0.0000	[-0.0011, 0.0010]	0.958	0.994
Regional volume	Fluid	Right pars orbitalis	1611	-0.0000	[-0.0011, 0.0009]	0.986	0.994
Regional volume	Fluid	Right rostral middle frontal gyrus	1611	-0.0000	[-0.0009, 0.0011]	0.992	0.994
Regional volume	Fluid	Left fusiform gyrus	1611	-0.0000	[-0.0010, 0.0010]	0.994	0.994

2.6 Key mediation sensitivity specifications

Right fornix FA remained significant for fluid cognition when adiposity was omitted, when waist circumference was added, and when ethnicity was included. Left parahippocampal volume produced small positive indirect estimates in the targeted sensitivity models, but it did not survive the full all-volume correction and is considered secondary.

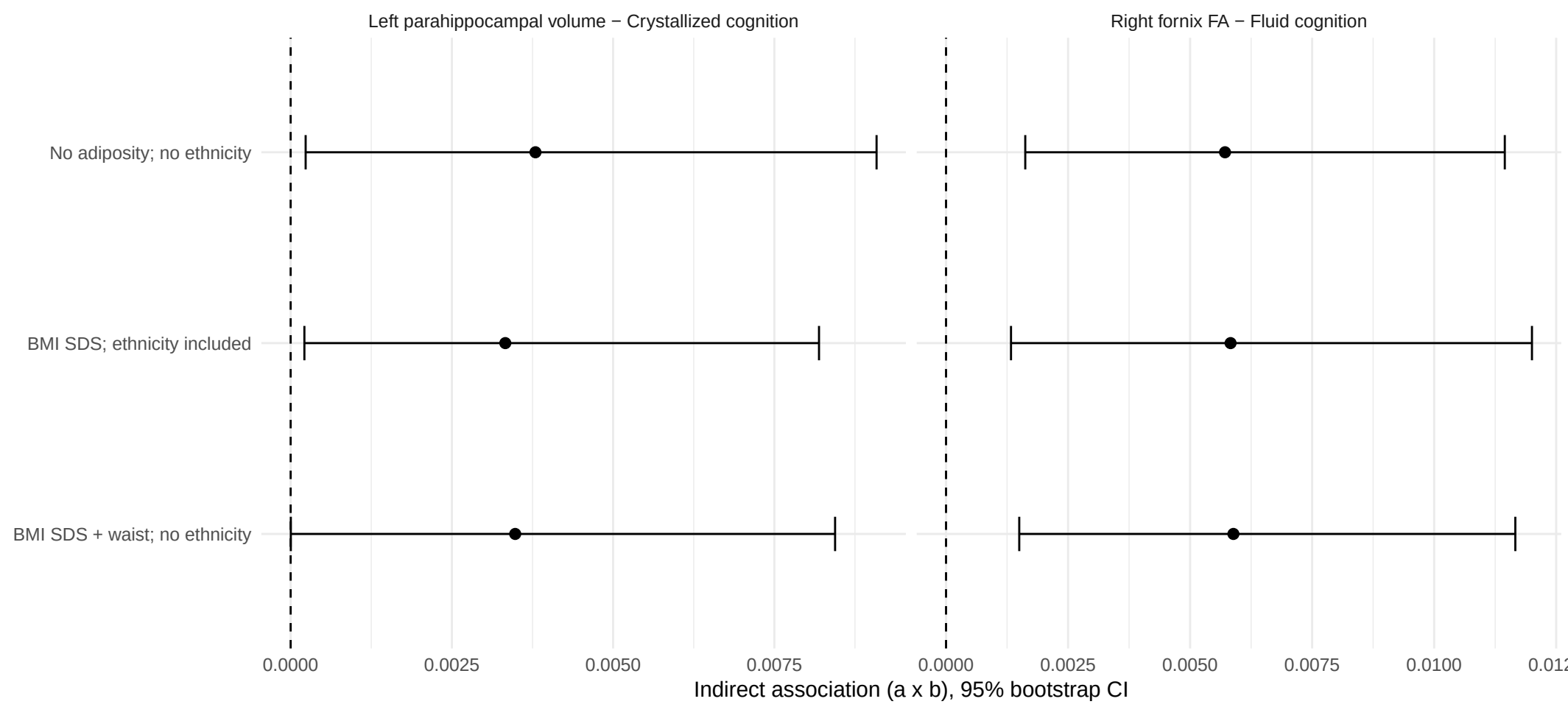

Figure S9: Right-fornix and left-parahippocampal indirect effects across sensitivity specifications.

Table S17: Sensitivity analyses for indirect associations involving right fornix FA and left parahippocampal volume.

Modality	Outcome	Mediator	Effect	N	Estimate	Bootstrap SE	95% CI	P	P_{FDR}
**Specification 1: No adiposity adjustment; ethnicity excluded**									
FA	Fluid cognition	Right fornix	a	1435	0.0844	0.0266	[0.0338, 0.1375]	< 0.001	< 0.001
FA	Fluid cognition	Right fornix	b	1435	0.0678	0.0191	[0.0288, 0.1046]	0.002	0.002
FA	Fluid cognition	Right fornix	Indirect	1435	0.0057	0.0025	[0.0016, 0.0114]	0.002	0.002
FA	Fluid cognition	Right fornix	Direct	1435	0.0571	0.0206	[0.0148, 0.0968]	0.008	0.008
FA	Fluid cognition	Right fornix	Total	1435	0.0628	0.0206	[0.0192, 0.1019]	0.002	0.002
FA	Fluid cognition	Right fornix	Proportion	1435	0.0910	0.1186	[0.0234, 0.3034]	0.004	0.004
Volume	Crystallized cognition	Left parahippocampal gyrus	a	1627	0.0579	0.0267	[0.0066, 0.1093]	0.034	0.034
Volume	Crystallized cognition	Left parahippocampal gyrus	b	1627	0.0655	0.0216	[0.0230, 0.1068]	0.006	0.006
Volume	Crystallized cognition	Left parahippocampal gyrus	Indirect	1627	0.0038	0.0023	[0.0002, 0.0091]	0.040	0.040
Volume	Crystallized cognition	Left parahippocampal gyrus	Direct	1627	0.0785	0.0211	[0.0391, 0.1207]	< 0.001	< 0.001
Volume	Crystallized cognition	Left parahippocampal gyrus	Total	1627	0.0823	0.0210	[0.0434, 0.1248]	< 0.001	< 0.001
Volume	Crystallized cognition	Left parahippocampal gyrus	Proportion	1627	0.0461	0.0367	[0.0028, 0.1394]	0.040	0.040
**Specification 2: BMI SDS and waist circumference; ethnicity excluded**									
FA	Fluid cognition	Right fornix	a	1413	0.0865	0.0276	[0.0324, 0.1382]	< 0.001	< 0.001
FA	Fluid cognition	Right fornix	b	1413	0.0681	0.0195	[0.0297, 0.1077]	< 0.001	< 0.001
FA	Fluid cognition	Right fornix	Indirect	1413	0.0059	0.0026	[0.0015, 0.0117]	< 0.001	< 0.001
FA	Fluid cognition	Right fornix	Direct	1413	0.0552	0.0197	[0.0174, 0.0951]	0.004	0.004
FA	Fluid cognition	Right fornix	Total	1413	0.0611	0.0197	[0.0233, 0.1009]	< 0.001	< 0.001
FA	Fluid cognition	Right fornix	Proportion	1413	0.0963	0.1312	[0.0245, 0.2911]	< 0.001	< 0.001

Continued on next page

Table S17 – continued

**Modality**	**Outcome**	**Mediator**	**Effect**	N	**Estimate**	**Bootstrap SE**	**95% CI**	P	P_{FDR}
Volume	Crystallized cognition	Left parahippocampal gyrus	a	1599	0.0572	0.0265	[0.0022, 0.1107]	0.042	0.042
Volume	Crystallized cognition	Left parahippocampal gyrus	b	1599	0.0609	0.0223	[0.0187, 0.1045]	0.008	0.008
Volume	Crystallized cognition	Left parahippocampal gyrus	Indirect	1599	0.0035	0.0022	[0.0000, 0.0084]	0.050	0.050
Volume	Crystallized cognition	Left parahippocampal gyrus	Direct	1599	0.0788	0.0213	[0.0402, 0.1214]	< 0.001	< 0.001
Volume	Crystallized cognition	Left parahippocampal gyrus	Total	1599	0.0823	0.0212	[0.0430, 0.1260]	< 0.001	< 0.001
Volume	Crystallized cognition	Left parahippocampal gyrus	Proportion	1599	0.0423	0.0340	[0.0001, 0.1367]	0.050	0.050
**Specification 3: BMI SDS adjustment; ethnicity included**									
FA	Fluid cognition	Right fornix	a	1423	0.0844	0.0274	[0.0330, 0.1386]	0.002	0.002
FA	Fluid cognition	Right fornix	b	1423	0.0690	0.0204	[0.0283, 0.1069]	0.004	0.004
FA	Fluid cognition	Right fornix	Indirect	1423	0.0058	0.0027	[0.0013, 0.0120]	0.006	0.006
FA	Fluid cognition	Right fornix	Direct	1423	0.0495	0.0196	[0.0101, 0.0888]	0.018	0.018
FA	Fluid cognition	Right fornix	Total	1423	0.0553	0.0197	[0.0163, 0.0939]	0.010	0.010
FA	Fluid cognition	Right fornix	Proportion	1423	0.1054	0.2633	[0.0231, 0.3790]	0.016	0.016
Volume	Crystallized cognition	Left parahippocampal gyrus	a	1611	0.0570	0.0260	[0.0068, 0.1058]	0.032	0.032
Volume	Crystallized cognition	Left parahippocampal gyrus	b	1611	0.0584	0.0213	[0.0179, 0.1014]	0.004	0.004
Volume	Crystallized cognition	Left parahippocampal gyrus	Indirect	1611	0.0033	0.0020	[0.0002, 0.0082]	0.032	0.032
Volume	Crystallized cognition	Left parahippocampal gyrus	Direct	1611	0.0714	0.0211	[0.0295, 0.1113]	< 0.001	< 0.001
Volume	Crystallized cognition	Left parahippocampal gyrus	Total	1611	0.0748	0.0212	[0.0321, 0.1150]	< 0.001	< 0.001

Continued on next page

Table S17 – continued

**Modality**	**Outcome**	**Mediator**	**Effect**	N	**Estimate**	**Bootstrap SE**	**95% CI**	P	P_{FDR}
Volume	Crystallized cognition	Left parahippocampal gyrus	Proportion	1611	0.0445	0.0394	[0.0024, 0.1452]	0.032	0.032

Note: The dietary exposure is the cross-fitted PolyIQ score corresponding to the cognitive outcome shown in each row. a denotes the diet–brain association; b denotes the brain–cognition association conditional on diet. The indirect association is the product ab. Direct denotes the diet–cognition association conditional on the brain measure, and Total is the sum of the direct and indirect associations. Confidence intervals and P values are based on 1,000 bootstrap replications. FA = fractional anisotropy; BMI SDS = age- and sex-standardized body mass index.

2.7 PC-based FA mediation

The PC analysis tested each dietary PC as the focal exposure while controlling for the remaining four. PC1 $\rightarrow$ right fornix FA $\rightarrow$ fluid cognition survived correction across all PC-by-tract indirect-effect tests. The corresponding crystallized result had an adjusted $P = 0.070$. No other PC-by-tract indirect effect survived the broad correction.

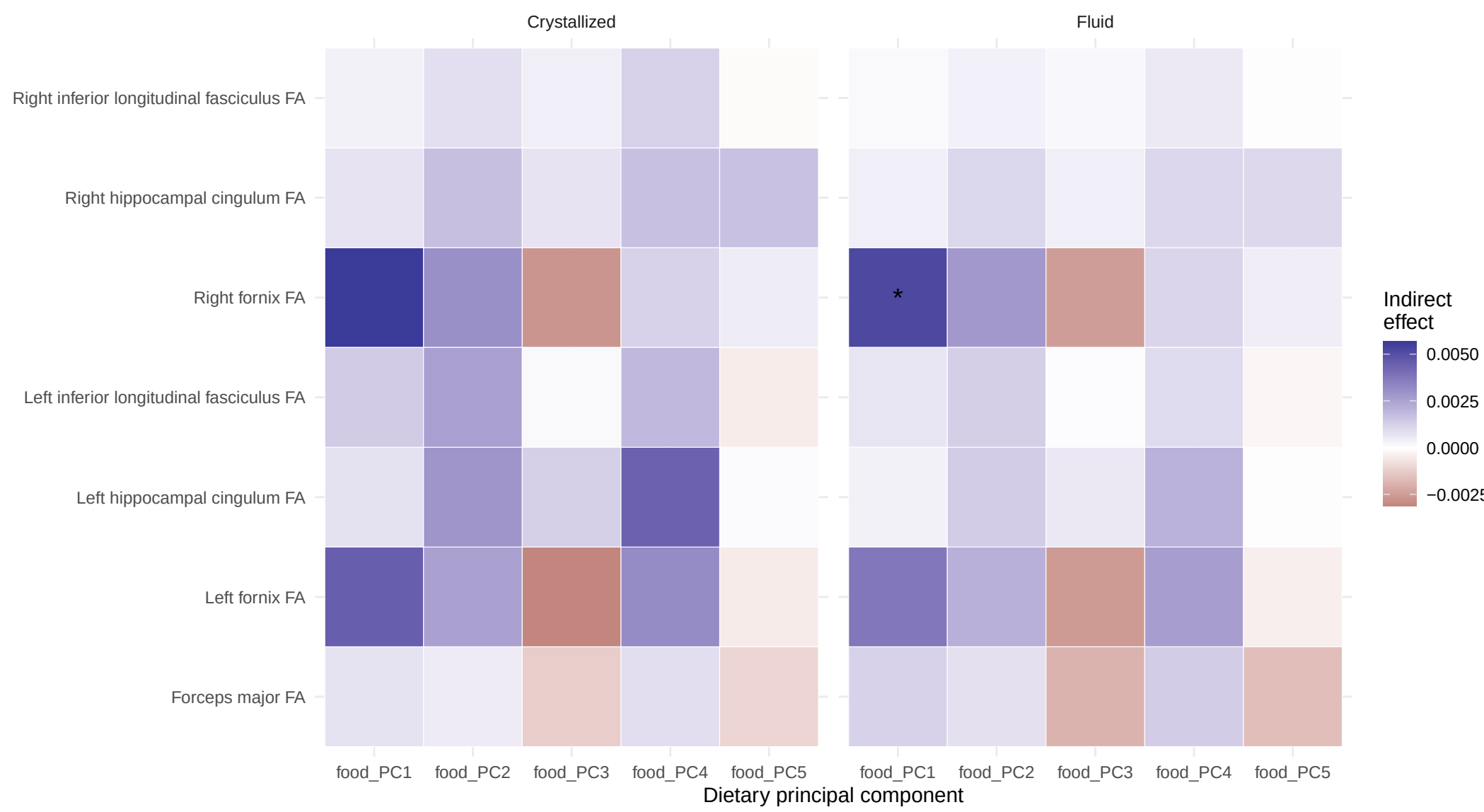

Figure S10: PC-based FA indirect-effect estimates and corrected significance.

2.8 Direct diet–brain models

Only the association of food PC1 with right fornix FA survived FDR correction. No individual raw food variable survived correction for right fornix FA, and no direct food-PC or raw-food association survived correction for left parahippocampal volume. The earlier interpretation centered on specific individual foods has therefore been removed.

BrainOutcome	DietaryPredictor	N	estimate	se	statistic	p_value	P_FDR
fxrh	PC1	1420	0.0010	0.0004	2.7546	0.0060	0.0298
fxrh	PC2	1420	0.0007	0.0005	1.5701	0.1166	0.1944
fxrh	PC3	1420	-0.0010	0.0006	-1.6201	0.1054	0.1944
fxrh	PC4	1420	0.0004	0.0007	0.6108	0.5414	0.6768
fxrh	PC5	1420	0.0000	0.0007	0.0411	0.9673	0.9673
fxrh	Fruits	1420	0.0019	0.0020	0.9689	0.3327	0.8412
fxrh	Vegetables	1420	0.0024	0.0034	0.7248	0.4687	0.8412
fxrh	Potato	1420	0.0002	0.0054	0.0426	0.9660	0.9660
fxrh	Whole Grain	1420	0.0050	0.0029	1.7442	0.0814	0.8135
fxrh	Meat/Poultry/Fish	1420	0.0006	0.0017	0.3859	0.6996	0.8412
fxrh	Dairy	1420	0.0015	0.0022	0.6768	0.4986	0.8412
fxrh	Legumes	1420	0.0029	0.0095	0.3093	0.7571	0.8412
fxrh	Sugary Beverages	1420	-0.0023	0.0068	-0.3372	0.7360	0.8412
fxrh	Soft Drinks	1420	0.0018	0.0056	0.3226	0.7471	0.8412
fxrh	Ketchup and salsa	1420	-0.0005	0.0009	-0.5558	0.5784	0.8412
LParaHippo	PC1	1608	0.8897	4.5521	0.1954	0.8451	0.8451
LParaHippo	PC2	1608	4.7131	5.7705	0.8168	0.4142	0.5177
LParaHippo	PC3	1608	-19.7056	8.1811	-2.4087	0.0161	0.0806
LParaHippo	PC4	1608	14.5808	8.2616	1.7649	0.0778	0.1642
LParaHippo	PC5	1608	14.5502	8.8022	1.6530	0.0985	0.1642
LParaHippo	Fruits	1608	-35.1807	24.4321	-1.4399	0.1501	0.3002
LParaHippo	Vegetables	1608	77.4974	42.8773	1.8074	0.0709	0.2186
LParaHippo	Potato	1608	8.8372	67.5849	0.1308	0.8960	0.9975
LParaHippo	Whole Grain	1608	98.0293	36.6538	2.6745	0.0076	0.0756
LParaHippo	Meat/Poultry/Fish	1608	-35.7761	20.9206	-1.7101	0.0874	0.2186
LParaHippo	bDairy	1608	14.7686	27.6062	0.5350	0.5927	0.9879
LParaHippo	Legumes	1608	-12.9916	116.5643	-0.1115	0.9113	0.9975
LParaHippo	Sugary Beverages	1608	0.2719	86.0125	0.0032	0.9975	0.9975
LParaHippo	Soft Drinks	1608	1.0188	70.9007	0.0144	0.9885	0.9975
LParaHippo	Ketchup and Salsa	1608	-25.1179	10.9563	-2.2926	0.0220	0.1100

Table S18: Direct dietary associations with right fornix FA and left parahippocampal volume.

3 Supplementary Additional Analyses

3.1 Nutrient-based robustness

The nutrient PCA recovered a strong general-intake component and additional contrasts involving glycemic index, fiber, added sugar, and fat. Food- and nutrient-based scores were correlated at $r = 0.721$ for crystallized and $r = 0.782$ for fluid cognition. Nutrient elastic-net and ordinary-regression scores were almost identical. For right fornix FA, nutrient PC4 was negatively associated after FDR correction; nutrient PC1 was positive but narrowly missed correction. No individual raw nutrient survived correction, although added sugar was nominally negative.

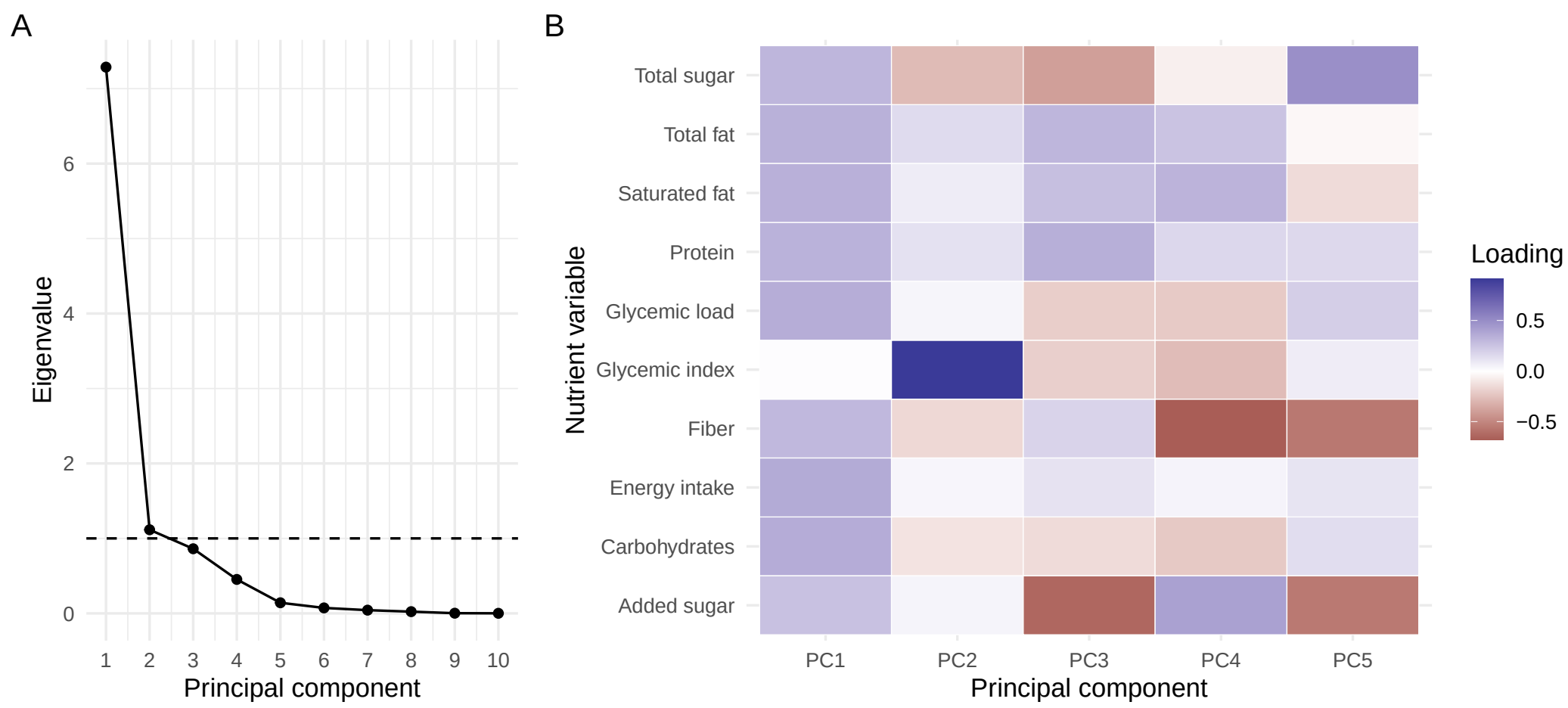

Figure S11: Nutrient PCA scree plot and loading heat map.

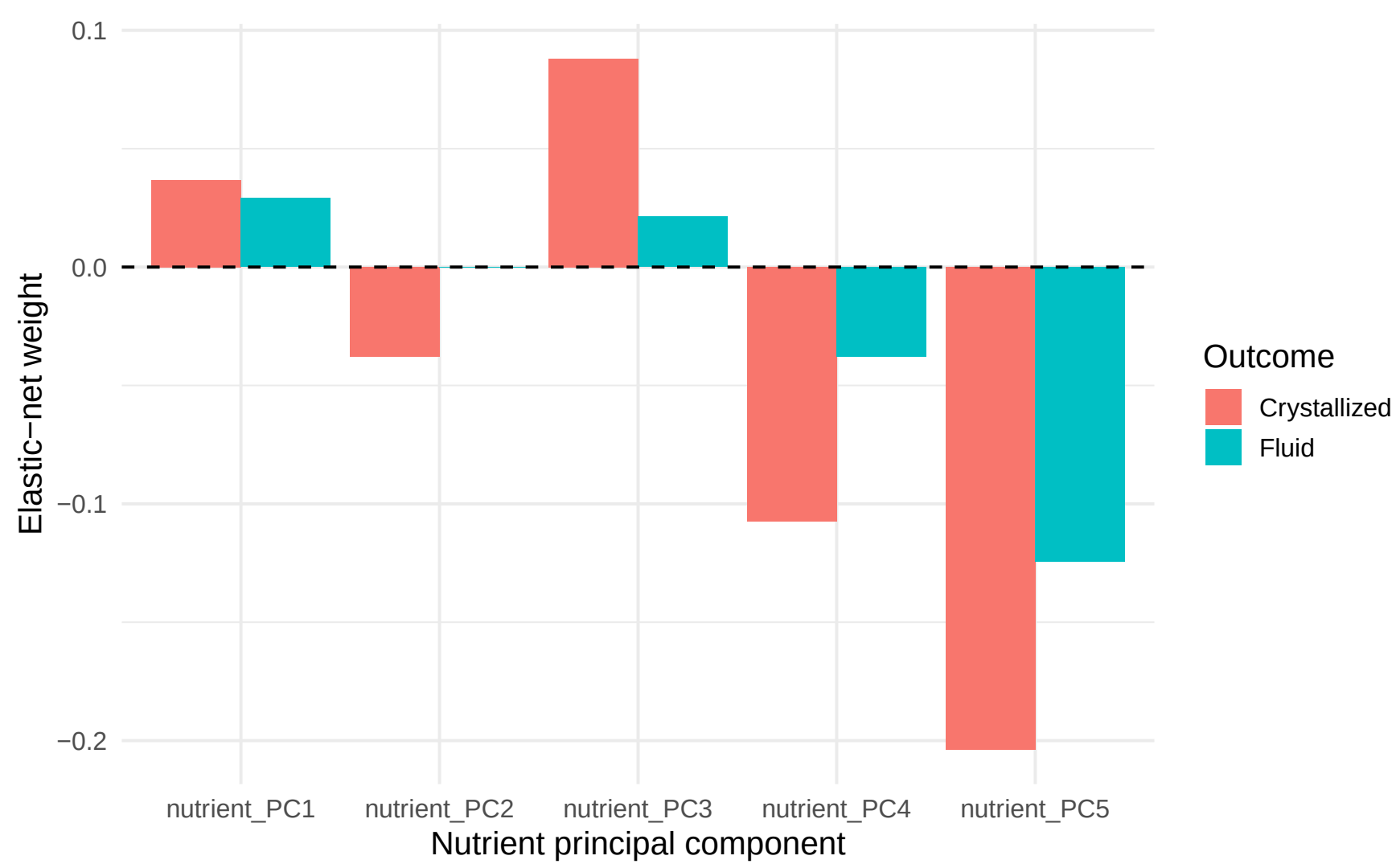

Figure S12: Elastic-net weights for nutrient-based cognition-aligned scores.

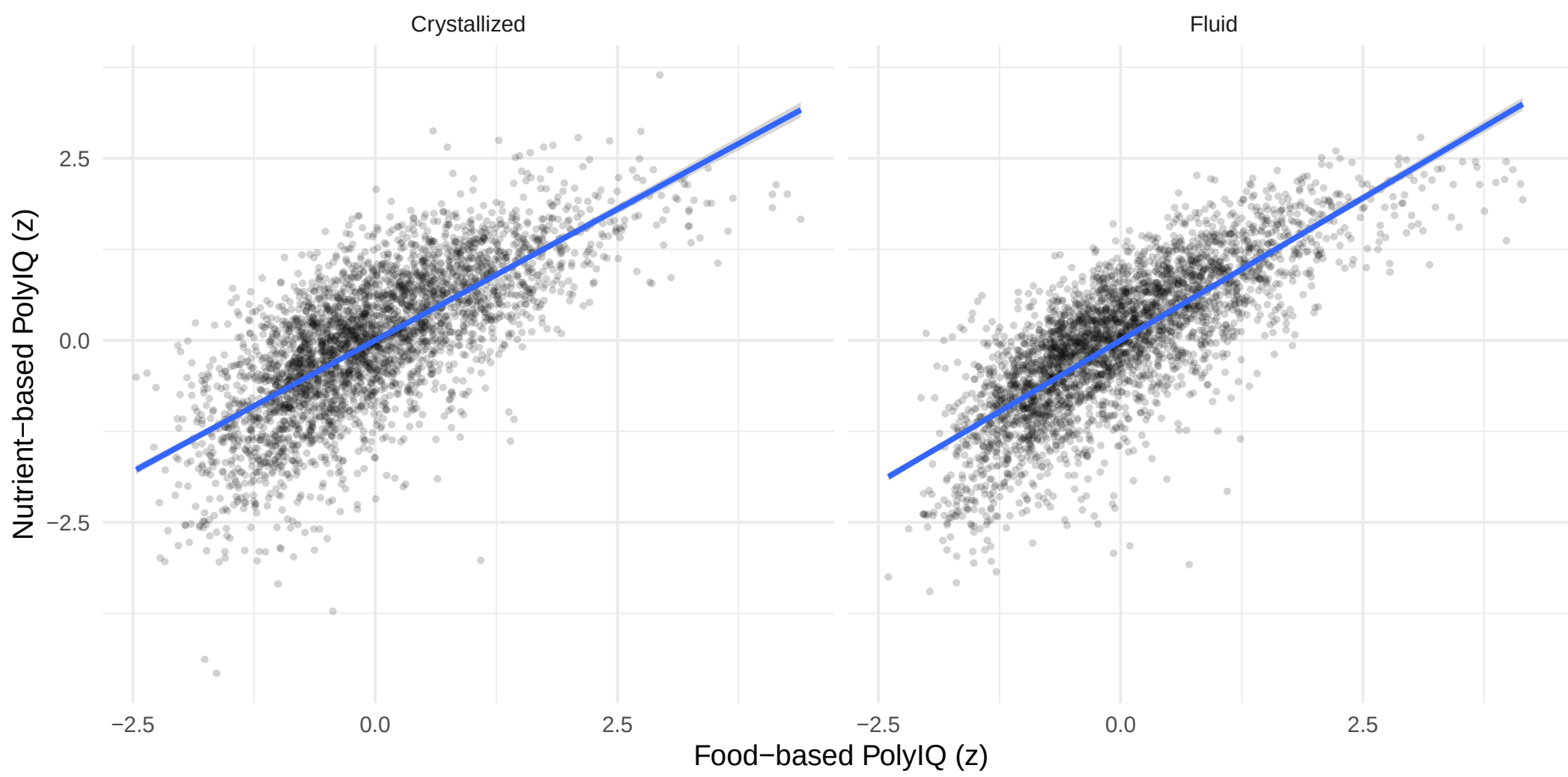

Figure S13: Food-based versus nutrient-based cognition-aligned scores.

PC	Eigenvalue	Proportion	Cumulative
PC1*	7.2870	0.7287	0.7287
PC2*	1.1157	0.1116	0.8403
PC3*	0.8619	0.0862	0.9265
PC4*	0.4528	0.0453	0.9717
PC5*	0.1415	0.0141	0.9859
PC6*	0.0726	0.0073	0.9931
PC7*	0.0428	0.0043	0.9974
PC8*	0.0229	0.0023	0.9997
PC9*	0.0021	0.0002	0.9999
PC10*	0.0007	0.0001	1.0000

NutrientVariable	PC1*	PC2*	PC3*	PC4*	PC5*
Carbohydrates	0.3583	-0.1112	-0.1430	-0.2175	0.1465
Protein	0.3342	0.1285	0.3483	0.1698	0.1661
Fiber	0.3074	-0.1563	0.1861	-0.6753	-0.5610
KCal	0.3663	0.0401	0.1216	0.0485	0.1150
Total fat	0.3398	0.1528	0.3134	0.2582	-0.0303
Sugar	0.3161	-0.2763	-0.3928	-0.0629	0.4915
Saturated fat	0.3411	0.0811	0.2767	0.3268	-0.1439
GI	0.0110	0.9136	-0.1937	-0.2702	0.0785
GL	0.3565	0.0433	-0.1987	-0.2134	0.2105
Sugar added	0.2689	0.0469	-0.6346	0.4117	-0.5561

FoodPC	PC1*	PC2*	PC3*	PC4*	PC5*
PC1	0.9022	-0.0441	0.1020	-0.0927	-0.0677
PC2	0.0235	-0.0301	0.5643	-0.4061	0.0530
PC3	-0.1540	0.1358	0.3420	0.2704	-0.1678
PC4	0.0069	-0.2836	0.1161	-0.0202	-0.2528
PC5	0.0851	0.4467	0.0818	0.4032	-0.0718

Table S19: Correlations between food-based and nutrient-based (noted with *) principal components.

Comparison	Correlation
Crystallized nutrient EN vs OLS	1.0000
Fluid nutrient EN vs OLS	0.9990
Crystallized food vs nutrient EN	0.7207
Fluid food vs nutrient EN	0.7821

Table S20: Comparisons of food-based and nutrient-based PolyIQ scores.

BrainOutcome	DietaryPredictor	N	estimate	se	statistic	p_value	P_FDR
fxrh	PC1*	1420	0.0005	0.0002	2.2924	0.0220	0.0551
fxrh	PC2*	1420	0.0002	0.0006	0.3803	0.7038	0.7038
fxrh	PC3*	1420	0.0003	0.0007	0.5278	0.5977	0.7038
fxrh	PC4*	1420	-0.0026	0.0009	-2.8184	0.0049	0.0245
fxrh	PC5*	1420	0.0014	0.0017	0.8446	0.3985	0.6641
fxrh	Carbohydrates	1420	0.0249	0.0322	0.7729	0.4397	0.4989
fxrh	Proteins	1420	0.0055	0.0054	1.0179	0.3089	0.4989
fxrh	Fiber	1420	0.0029	0.0038	0.7573	0.4490	0.4989
fxrh	KCal	1420	-0.0324	0.0222	-1.4635	0.1436	0.4786
fxrh	Tota Fat	1420	0.0085	0.0096	0.8812	0.3784	0.4989
fxrh	Sugar	1420	0.0076	0.0040	1.8862	0.0595	0.2974
fxrh	Saturated fat	1420	0.0043	0.0055	0.7903	0.4295	0.4989
fxrh	GI	1420	0.0378	0.0307	1.2312	0.2185	0.4989
fxrh	GL	1420	-0.0139	0.0295	-0.4699	0.6385	0.6385
fxrh	Sugar added	1420	-0.0040	0.0019	-2.0764	0.0380	0.2974

Table S21: Nutrient-based dietary associations with right fornix FA.

3.2 Moderation of the association between cognition-aligned dietary patterns and cognitive performance

We examined whether the association between the cross-fitted cognition-aligned dietary score and cognitive performance varied with SJL, pubertal stage, SES, or INR. These analyses used the same primary covariate specification as the revised cognition models, including BMI SDS but excluding ethnicity, and included a family-level random intercept. Because the dietary scores were standardized before analysis, interaction coefficients refer to the change in the cognition–PolyIQ association associated with a one-standard- deviation change in the moderator. The four interaction tests within each cognitive domain were additionally corrected using the Benjamini–Hochberg procedure. We found that INR significantly moderated the association between the fluid cognition-aligned dietary score and fluid cognition ($\beta = -0.054$, SE = 0.017, $p = .002$, $p_{\mathrm{FDR}} = .006$). The negative interaction indicates that the diet–cognition association was stronger at lower levels of INR and diminished as INR increased. No other interaction survived FDR correction. For crystallized cognition, interactions with SJL, pubertal stage, and INR were suggestive at the uncorrected level but did not survive multiple-comparison correction (all $p_{\mathrm{FDR}} = .082$).

3.3 Socioeconomic position, cognition-aligned dietary patterns, and cognitive performance

We also examined whether the association of SES and INR with cognitive performance was statistically accompanied by an indirect association through the cognition-aligned dietary score. To maintain consistency with the revised analyses, we used the family-level cross-fitted PolyIQ scores and the primary covariate specification. Uncertainty in the indirect association was estimated using a family-cluster bootstrap with 1,000 replications. Because the data are cross-sectional and PolyIQ is a supervised, cognition-aligned dietary summary, these estimates are interpreted as decompositions of shared covariance rather than evidence that nutrition causally mediates socioeconomic differences in cognition.

Significant positive indirect associations were observed for both socioeconomic measures and both cognitive domains. For crystallized cognition, the indirect association was 0.021 (95% CI [0.013, 0.031], $p_{\mathrm{FDR}} = .002$) for SES and 0.018 (95% CI [0.011, 0.026], $p_{\mathrm{FDR}} = .002$) for INR, corresponding to approximately 8.9% and 8.0% of the respective total associations. For fluid cognition, the corresponding indirect associations were 0.012 (95% CI [0.006, 0.019], $p_{\mathrm{FDR}} = .002$) for SES and 0.011 (95% CI [0.006, 0.017], $p_{\mathrm{FDR}} = .002$) for INR, corresponding to approximately 7.0% and 7.8% of the total associations.

3.4 Outcome-independent dietary PC analysis of socioeconomic indirect associations

As an outcome-independent robustness analysis, we repeated the socioeconomic pathway analysis using each of the five unsupervised dietary principal components in place of PolyIQ. Because the dietary PCs were constructed exclusively from dietary variables and without using cognitive outcomes, these models provide a complementary test that does not rely on cognition-informed weighting of the dietary mediator.

For each socioeconomic variable (SES and income-to-needs ratio), we estimated separate indirect-association models through PC1–PC5 for crystallized and fluid cognition. Models used the same primary covariate specification as the revised analyses, including BMI SDS and excluding ethnicity, with a family-level random intercept. Confidence intervals and P values were obtained using a family-cluster bootstrap with 1,000 replications. We corrected the five indirect-effect tests within each socioeconomic-variable–cognition combination using the Benjamini–Hochberg procedure and additionally report a more conservative correction across all 20 tests.

For crystallized cognition, significant positive indirect associations were observed through both PC1 and PC2 for SES and INR. All four associations survived correction across the five PCs within each socioeconomic model and also the more conservative correction across all 20 PC-based tests (all $p_{\mathrm{FDR},20} = .007$). For fluid cognition, significant indirect associations were concentrated in PC1: the indirect association was 0.008 (95% CI [0.003, 0.013]) for SES and 0.007 (95% CI [0.003, 0.011]) for INR, with both surviving correction across all 20 tests ($p_{\mathrm{FDR},20} = .007$). No other PC-based indirect association with fluid cognition survived correction.

Thus, the socioeconomic pattern observed with the cross-fitted cognition-aligned dietary scores was also detectable using dietary components constructed independently of cognitive outcomes. The results further suggest that the association is distributed across PC1 and PC2 for crystallized cognition, whereas the corresponding association with fluid cognition is concentrated primarily in PC1.
